# Accelerometer-based physical activity, air pollution and risk of dementia subtypes: a prospective study using UK biobank

**DOI:** 10.3389/fpubh.2025.1695462

**Published:** 2026-01-13

**Authors:** Yiming Zhang, Wei Xu

**Affiliations:** 1 Department of Geography, University of Wisconsin-Milwaukee, Milwaukee, WI, United States; 2 Division of Epidemiology and Social Sciences, Institute for Health and Humanity, Medical College of Wisconsin, Milwaukee, WI, United States

**Keywords:** accelerometer, air pollution, Alzheimer’s disease, physical activity, vascular dementia

## Abstract

**Background:**

The protective effect of physical activity on dementia risk is well established, yet the magnitude of effect remains highly inconsistent, possibly due to various approaches to physical activity measurement and potential moderating effects of environmental factors such as air pollution.

**Objective:**

To evaluate the independent and interaction effects of air pollution and accelerometer-based physical activity on incident risks of dementia subtypes.

**Methods:**

This study included 96,661 participants from the UK Biobank study with accelerometer-derived physical activity measures. Dementia diagnosis was algorithmically defined based on self-reports, primary care, hospital, and mortality records. We assessed five air pollutants, including particulate matters (PM_2.5_, PM_2.5–10_, PM_10_), nitrogen dioxide (NO_2_), and nitrous oxides (NO_x_). Multivariable-adjusted Cox proportional hazard models were employed to estimate the associations between physical activity, air pollution, and the risks of Alzheimer’s Disease (AD) and Vascular Dementia (VaD). Hazard ratios (HRs) and 95% confidence intervals (CIs) were calculated. Several sensitivity analyses were conducted to assess the robustness of the results.

**Results:**

A one-IQR (10.13 milli-gravity) higher level of physical activity was significantly associated with a 30% lower risk of AD (HR = 0.7, 95% CI: 0.57–0.86, *p* < 0.001) and a 32% lower risk of VaD (HR = 0.68, 95% CI: 0.5–0.91, *p* < 0.05). Exposure to NO_2_ was significantly associated with an increased incident risk of AD, while exposure to PM_2.5_, PM_2.5–10_, PM_10_, and NO_2_ was associated with an increased risk of VaD. Results from models with interaction terms indicate that the protective effect of physical activity against AD was attenuated among individuals exposed to higher levels of PM_2.5_ and NO_2,_ and that the effect of physical activity against VaD was attenuated by higher PM_2.5–10_ exposure.

**Significance:**

This study provides new evidence on the independent and interactive effects of objectively measured physical activity and air pollution on the risks of dementia subtypes, thereby improving the biological specificity of these associations.

## 1 Introduction

Dementia refers to a broad category of conditions that impair memory, cognitive abilities, and behavior, causing considerable challenges in maintaining daily living activities (1). The World Health Organization reported that over 55 million individuals were living with dementia worldwide as of 2021, with about 10 million new cases occurring each year (2). Given the enormous social and economic costs dementia inflicts on individuals, their families, and society as a whole (3), it is critical to formulate effective public health strategies to reduce the disease burden at the population level. The lack of progress in identifying curative treatments for dementia has led scientists and public health agencies to shift their attention to prevention.

Physical activity has been recognized as one of the most significant protective factors against incident dementia by the Lancet Commission on Dementia (4). However, several longitudinal studies have also found that physical activity does not effectively reduce the risk of incident dementia (5–7), and systematic reviews have concluded that the heterogeneity in the effect size of physical activity on dementia risk among studies is high (8). The reasons behind the inconsistency in the associations between physical activity and dementia risk across studies are multifold. One potential contributing factor is the variations in how physical activity is measured. Broadly, physical activity measurements can be categorized into subjective and objective approaches. Subjective measurements of physical activity rely on self-reports, typically through surveys or questionnaires such as the International Physical Activity Questionnaire, the Saltin-Grimby Physical Activity Level Scale, or the Paffenbarger Physical Activity Questionnaire (9–12). However, researchers have questioned the reliability and validity of these recall-based physical activity measurements. A study using data from 12 countries found that the criterion validity of subjective physical activity measures based on the International Physical Activity Questionnaire showed a weak correlation of approximately 0.3 with accelerometer-based measurements (13). Furthermore, factors such as region (urban or rural), culture, education, and population characteristics were found to impact the reliability of subjective physical activity measurements (13–15). In contrast, physical activity measurements based on actigraphy, such as accelerometers, provide more objective measurements of physical activity levels and patterns (16, 17). Compared to subjective measures, objective PA measures have been shown to better predict the risk of various health outcomes, such as cardiovascular diseases (18–20) and mortality (21–23). Therefore, how physical activity is measured may be contributing to the inconsistency in its associations with dementia risk across studies.

Secondly, the association between physical activity and dementia risk may vary by dementia subtypes. Common dementia subtypes include Alzheimer’s Disease (AD), Vascular Dementia (VaD), Frontotemporal dementia (FTD), and Lewy body dementia, with AD and VaD being the most prevalent (50–75% of all dementia cases are AD, 17–30% are VaD) (24). Amyloid protein and neurofibrillary tangles are recognized as the dominant pathogenesis mechanisms of AD (25); VaD results from cerebrovascular diseases, particularly following a stroke; similarly to AD, FTD involves brain cell damage, but the affected regions are more localized to the frontal and temporal lobes; and Lewy body dementia is characterized by the presence of abnormal protein deposits (Lewy bodies) in brain nerve cell (24). Physical activity may be implicated in the risk of developing these diseases in different ways due to distinct etiologies underlying them. In a systematic review of studies on physical activity and risk of neurodegenerative diseases, Hamer, M. and Y. Chida (26) showed that the highest level of physical activity was associated with a greater reduced risk of AD than for all-cause dementia. Another meta-analysis of prospective studies reported that leisure-time physical activity was associated with reduced risks of all-cause dementia and AD but not for VaD, and that the beneficial effect of physical activity was slightly greater for AD than for all-cause dementia (27).

Thirdly, the inconsistency may be attributable to the potential moderating effects of other factors, such as air pollution, that may not be captured across studies. Accumulating evidence suggests that exposure to particulate matters (e.g., PM_2.5_ (28–35), PM_2.5–10_ (29), and PM_10_ (30, 33, 35)) and gaseous pollutants (e.g., ozone (28, 31, 32), NO_2_ (28, 31, 35, 36), and NO_x_ (33, 37)) may increase the risk of incident dementia. For example, a longitudinal study conducted in Northern Sweden with 1,806 participants found that PM_2.5_ from traffic exhaust and residential wood burning were both associated with an increased risk of all-cause dementia (38). A population-based study involving approximately 2.1 million adults in Canada also found that exposures to PM_2.5_ and NO_2_ were both associated with increased incidence of all-cause dementia (28); however, no significant association was observed with exposure to ozone. Based on nearly half a million middle-aged individuals, Yuan, S., et al. (33) found that those exposed to a higher level of PM_2.5_ (≥10 μg/m3) had a greater risk of all-cause dementia (but not AD and VaD) and those exposed to a higher level of NO_x_ (≥50 μg/m3) showed increased risks of both all-cause dementia and AD. Despite the known protective effect of physical activity against dementia risk, there is limited evidence on whether and to what extent exposure to air pollution negates this protective effect.

To address these gaps and improve the biological specificity in the associations between physical activity, air pollution exposures and dementia, this study used the large-scale longitudinal cohort data from the UK Biobank to evaluate the independent and interactive effects of five types of particulate matter and gaseous pollution exposures (i.e., PM_2.5_, PM_2.5–10_, PM_10_, NO_2_, NO_x_) and accelerometer-based physical activity measures on the incident risk of dementia subtypes, including AD and VaD.

## 2 Data and methods

### 2.1 Data

**Figure 1:**
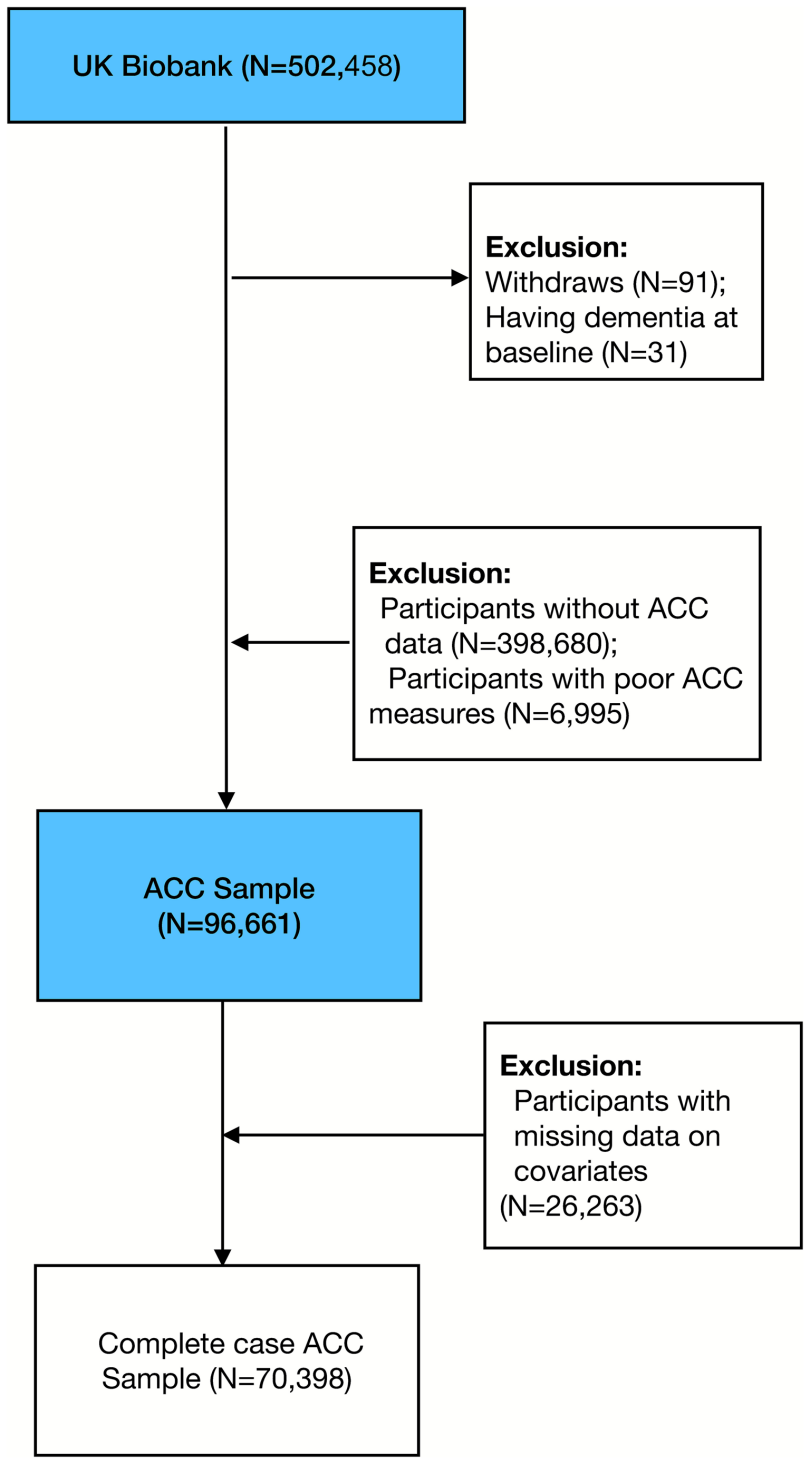
Flowchart of study participants selected from the UK Biobank.

The data used in this study is derived from UK Biobank, a large prospective study designed to understand the causes of a wide range of complex diseases of middle and old age (39). The UK Biobank’s ethical approval was granted by the National Information Governance Board for Health and Social Care and the NHS North West Multicenter Research Ethics Committee (REC) (REC reference: 16/NW/0274). From 2006 to 2010, over 500,000 individuals aged 40–69 across the UK participated in the study (39), with over 100,000 participants enrolled in the accelerometer study (40). UK Biobank collects a wide range of sociodemographic, behavioral, environmental, and health information, enabling comprehensive longitudinal health research. For our analysis, we excluded participants who withdrew from the UK Biobank study (*N* = 91), participants who had a dementia diagnosis at baseline (*N* = 31), and participants who did not enroll in the accelerometer study (*N* = 398,680). We further excluded those with low-quality accelerometer data, as indicated by quality score and wearing time (*N* = 6,995). This selection process is detailed in Figure 1, resulting in a final analytical sample of 96,661 participants. In the complete case analysis, we only included participants with no missing values for the covariates, reducing the sample size to 70,398.

### 2.2 Dementia diagnosis

We used algorithmically defined dementia outcomes (fields 42,020, 4,202, 42,022, and 42,023) in the UK Biobank for dementia incidence. The algorithm derived dementia diagnoses from a combination of data sources, including primary care, hospital admissions, or mortality data, using predefined International Classification of Diseases (ICD) codes (see Supplementary eTables 1, 2 for the ICD-9 and ICD-10 codes for AD and VaD). Time to event was calculated from the date of the initial assessment to the date of the earliest relevant health event in the linked data (event). Data is right censored if the participant did not have an AD or VaD diagnosis at the end of the study (censored), or was lost to follow-up (censored). Loss to follow-up was due to participants moving out of the UK, withdrawing consent for future data linkage, or death. We only selected AD and VaD cases for further analysis, due to the small number of FTD cases (*N* = 15).

### 2.3 Physical activity measurement

UK Biobank participants were invited via email to wear a wrist-worn Axivity A × 3 triaxial accelerometer to capture their physical activity level for a 7-day period. Over 100,000 participants consented, received the device by mail, and were instructed to wear it on their wrists continuously and carry out their normal activities (40). The device recorded tri-axial acceleration data with a dynamic range of ±8 g at a sampling rate of 100 Hz, and physical activity was measured in milli-gravity units (mg). Non-wear time, defined as continuous stationary periods lasting at least 60 min with minimal movement, was systematically removed from the dataset. To maintain data integrity and minimize diurnal bias, these segments were imputed using the average physical activity levels from similar periods. The monitoring instrument and detailed data processing protocols are described in the UK Biobank documentation (40, 41), which recommends a minimum wear time of 72 h to ensure reliable measurements. We further refined data quality by requiring a wear time of at least 3 days and a quality score of 1, indicating optimal device usage. Additionally, we used average acceleration adjusted for no-wear time bias and retained values below 1,000 mg to ensure data quality. To enable comparisons across types of air pollution and dementia risk, physical activity measurements were standardized using their interquartile range (IQR) of 10.13 mg.

### 2.4 Residential air pollution

Annual air pollution measures for PM_2.5_, PM_2.5–10_, PM_10_, NO_2_, and NO_x_ in 2010 were estimated for each participant’s address using a Land Use Regression (LUR) model developed within the European Study of Cohorts for Air Pollution Effects (ESCAPE) project (42, 43).^1^ The LUR model’s estimates were based on ESCAPE monitoring conducted from January 26, 2010 to January 18, 2011. These environmental exposure data were obtained from the Small Area Health Statistics Unit^2^ through the BioSHaRE-EU Environmental Determinants of Health Project,^3^ in which the UK Biobank participated (42, 43).

While previous studies have relied on average annual estimates of air pollution from 2005 to 2010 (44–47), we focused specifically on 2010 to avoid potential biases associated with combining estimates from different time periods and models. In the UK Biobank, pollution levels for 2005 to 2007 were estimated by an EU-wide model, which differs methodologically from the ESCAPE model. As noted by the UK Biobank, the pollution estimates from these two models are not directly comparable, and averaging them could introduce bias due to inconsistencies in measurement and modeling approaches (48). By using the 2010 estimates, we aimed to provide a more consistent and reliable assessment of air pollution exposure in the analysis of dementia risk.

### 2.5 Covariates

The models adjusted for the following covariates including age at the initial assessment (modeled as a continuous variable), sex (female or male), race (White, non-White), educational attainment (less than college, college or higher), smoking status (never, previous, current), family history of dementia in a parent or sibling (yes or no), history of stroke (yes or no), high blood pressure (yes or no), body mass index (BMI) [<25, (25–30), > = 30], and Townsend deprivation index, derived from the national census output area associated with the participant’s postcode, was included as a continuous variable. The statistical information is shown in Table 1.

**Table 1:** Descriptive statistics of baseline characteristics by AD and VaD.

Variables	AD			VaD		
	Non incident cases (*n* = 96,426)	Incident cases (*n* = 235)	*p*-value	Non incident cases (*n* = 96,549)	Incident cases (*n* = 112)	*p*-value
Age, y, mean(SD)	56.1 (7.82)	64.1 (4.38)	<0.001	56.1 (7.82)	63.9 (4.88)	<0.001
Sex			0.155			<0.001
Female	54,301 (56.3)	121 (51.5)		54,378 (56.3)	44 (39.3)	
Male	42,125 (43.7)	114 (48.5)		42,171 (43.7)	68 (60.7)	
Race			0.684			0.589
Non-White	2,977 (3.1)	5 (2.1)		2,980 (3.1)	2,980 (3.1)	
White	93,114 (96.6)	229 (97.4)		93,233 (96.6)	93,233 (96.6)	
Missing	335 (0.3)	1 (0.4)		336 (0.3)	0	
Education			<0.001			<0.001
Less than college	46,032 (47.7)	112 (47.7)		46,097 (47.7)	47 (42.0)	
College or higher	41,467 (43.0)	77 (32.8)		41,509 (43.0)	35 (31.2)	
Missing	8,927 (9.3)	46 (19.6)		8,943 (9.3)	30 (26.8)	
Smoking Status			0.002			<0.001
Never	54,946 (57.0)	111 (47.2)		55,009 (57.0)	48 (42.9)	
Previous	34,553 (35.8)	112(47.7)		34,604 (35.8)	61 (54.5)	
Current	6,670 (6.9)	11 (4.7)		6,678 (6.9)	3 (2.7)	
Missing	257 (0.3)	1 (0.4)		258 (0.3)	0	
Family history of dementia			<0.001			0.107
No	74,056 (76.8)	156 (66.4)		74,135 (76.8)	77 (68.8)	
Yes	10,433 (10.8)	50 (21.3)		10,465 (10.8)	18 (16.1)	
Missing	11,937 (12.4)	29 (12.3)		11,949 (12.4)	17 (15.2)	
History of Stroke			0.141			0.616
No	95,468 (99.0)	231 (98.3)		95,588 (99.0)	111 (99.1)	
Yes	816 (0.8)	4 (1.7)		819 (0.8)	1 (0.9)	
Missing	142 (0.1)	0		142 (0.1)	0	
High blood pressure			0.079			0.084
No	76,041 (78.9)	172 (73.2)		76,134 (78.9)	79 (70.5)	
Yes	20,243 (21.0)	63 (26.8)		20,273 (21.0)	33 (29.5)	
Missing	142 (0.1)	0		142 (0.1)	0	
Townsend deprivation, mean(SD)	−1.73 (2.82)	−1.62 (2.86)	0.573	−1.73 (2.82)	−1.66 (3.16)	0.831
BMI			0.241			0.836
< 25	37,870 (39.3)	94 (40.0)		37,922 (39.3)	42 (37.5)	
(25–30)	39,627 (41.1)	94 (40.0)		39,676 (41.1)	45 (40.2)	
> = 30	18,713 (19.4)	45 (19.1)		18,733 (19.4)	25 (22.3)	
Missing	216 (0.2)	2 (0.9)		218 (0.2)	0	
ACC, mg, mean(SD)	28.18 (8.41)	24.25 (7.56)	<0.001	28.18 (8.41)	23.88 (8.52)	<0.001
PM_2.5_, mean(SD)	9.9 (1.03)	9.97 (1.08)	0.328	9.9 (1.03)	9.92 (0.89)	0.809
PM_2.5__quartile			0.642			0.135
Q1 (8.17–9.20)	22,299 (23.1)	54 (23.0)		22,335 (23.1)	18 (16.1)	
Q2 (9.20–9.86)	22,296 (23.1)	57 (24.3)		22,317 (23.1)	36 (32.1)	
Q3 (9.86–10.48)	22,303 (23.1)	50 (21.3)		22,330 (23.1)	23 (20.5)	
Q4 (10.48–19.89)	22,292 (23.1)	61 (26.0)		22,328 (23.1)	25 (22.3)	
Missing	7,236 (7.5)	13 (5.5)		7,239 (7.5)	10 (8.9)	
PM_2.5–10_, mean(SD)	6.41 (0.89)	6.55 (1.05)	0.048	6.41 (0.89)	6.46 (0.97)	0.656
PM_2.5–10__quartile			0.16			0.12
Q1 (5.57–5.84)	22,297 (23.1)	56 (23.8)		22,337 (23.1)	16 (14.3)	
Q2 (5.84–6.10)	22,303 (23.1)	50 (21.3)		22,318 (23.1)	35 (31.2)	
Q3 (6.10–6.61)	22,306 (23.1)	47 (20.0)		22,328 (23.1)	25 (22.3)	
Q4 (6.61–12.82)	22,284 (23.1)	69 (29.4)		22,327 (23.1)	26 (23.2)	
Missing	7,136 (7.5)	13 (5.5)		7,239 (7.5)	10 (8.9)	
PM_10_, mean(SD)	16.17 (1.91)	16.41 (2.22)	0.104	16.17 (1.91)	16.41 (1.83)	0.178
PM_10__quartile			0.236			0.303
Q1 (11.78–15.18)	22,295 (23.1)	58 (24.7)		22,336 (23.1)	17 (15.2)	
Q2 (15.18–16.00)	22,298 (23.1)	55 (23.4)		22,326 (23.1)	27 (24.1)	
Q3 (16.00–16.97)	22,309 (23.1)	44 (18.7)		22,321 (23.1)	32 (28.6)	
Q4 (16.97–30.65)	22,288 (23.1)	65 (27.7)		22,327 (23.1)	26 (23.2)	
Missing	7,236 (7.5)	13 (5.5)		7,239 (7.5)	10 (8.9)	
NO_2_, mean(SD)	26.15 (7.61)	26.41 (7.87)	0.618	26.15 (7.61)	25.63 (6.42)	0.39
NO_2__quartile			0.327			0.006
Q1 (12.93–20.77)	23,799 (24.7)	48 (20.4)		23,825 (24.7)	22 (19.6)	
Q2 (20.77–25.65)	23,776 (24.7)	70 (29.8)		23,801 (24.7)	45 (40.2)	
Q3 (25.65–30.87)	23,789 (24.7)	57 (24.3)		23,823 (24.7)	23 (20.5)	
Q4 (30.87–106.43)	23,788 (24.7)	58 (24.7)		23,825 (24.7)	21 (18.8)	
Missing	1,274 (1.3)	2 (0.9)		1,275 (1.3)	1 (0.9)	
NO_x_, mean(SD)	42.93 (15.23)	43.1 (15.92)	0.87	42.93 (15.2)	42.95 (12.5)	0.982
NO_x_ _quartile			0.774			0.961
Q1 (19.74–33.30)	23,791 (24.7)	56 (23.8)		23,822 (24.7)	25 (22.3)	
Q2 (33.30–41.37)	23,783 (24.7)	63 (26.8)		23,816 (24.7)	30 (26.8)	
Q3 (41.37–49.77)	23,794 (24.7)	52 (22.1)		23,818 (24.7)	28 (25.0)	
Q4 (49.77–265.94)	23,784 (24.7)	62 (26.4)		23,818 (24.7)	28 (25.0)	
Missing	1,274 (1.3)	2 (0.9)		1,275 (1.3)	1 (0.9)	

ACC, accelerometer based total volume of physical activity in mg; AD, Alzheimer’s disease; BMI, body mass index; NO_2_, nitrogen dioxide; NO_x_, nitrogen oxides; PM, particulate matter; SD, standard deviation; VaD, Vascular Dementia. *p* values are based on observations without missing values.

Regarding missing data, proportions were relatively low: family history of dementia [11,966 (12.4%)], educational attainment [8,973 (9.3%)], PM_2.5_ [7,249 (7.5%)], PM_2.5–10_ [7,249 (7.5%)], PM_10_ [7,249 (7.5%)], NO_2_ [1,276 (1.3%)], NO_x_ [1,276 (1.3%)], race [336 (0.3%)], smoking status [258 (0.3%)], BMI [218 (0.2%)], history of stroke [142 (0.1%)], high blood pressure [142 (0.1%)], and Townsend deprivation index [110 (0.1%)]. To mitigate the missing values issue and maintain sample power, missing data for each variable were imputed using a standard multiple imputation procedure. This procedure considered the variable’s association with other covariates, assuming that the data were missing at random (MAR) (49). The standard multiple imputation procedure was performed using the mice package in R, and coefficient estimates from regression models were pooled from 5 imputed datasets according to Rubin’s Rule (49, 50). List and definitions of variables entered in the imputation models are detailed in Supplementary eTables 3, 4.

### 2.6 Statistical analysis

Cox proportional hazard models were estimated to examine the associations between different air pollution exposures, accelerometer-based physical activity, and the risk of dementia subtypes. The main model included measures of physical activity and air pollution exposures (without the interaction term of air pollution and physical activity) and adjusted for all covariates. HRs and 95% CIs were calculated to assess these associations. Accelerometer data were standardized by their IQR of 10.13 mg for comparability, and air pollutant associations were analyzed using quartiles. Interaction models were constructed to examine the potential interaction effects of exposure to each type of air pollution and accelerometer-based physical activity on the risk of dementia subtypes. Analyses were conducted using imputation datasets, and results were pooled. We further carried out several sensitivity analyses as robustness checks, including analyses using the complete case dataset (participants without missing values for all variables), analyses based on participants who did not receive a dementia diagnosis in the first 2 years of follow-up to mitigate possible reverse causality, and analyses based on participants who were aged 60 years and over at baseline to examine whether effects may be more pronounced among those who were older at baseline. Sensitivity analysis results are in the Supplementary. We also employed cubic splines to examine potential nonlinear associations between physical activity, air pollution, and dementia risk. However, we did not observe evidence of nonlinear relationships between physical activity and dementia risk across different air pollution levels. Because this is an exploratory study, we did not limit our ability to detect signals of associations by adjusting *p*-values for multiple comparisons (51).

## 3 Result

Descriptive statistics of the analytical sample (*N* = 96,661) are presented in Table 1. A total of 235 AD cases and 112 VaD cases were identified. Individuals diagnosed with AD were significantly older, had lower education levels, were more likely to have smoked, and had a family history of dementia. Participants diagnosed with VaD were significantly older, more likely to be male, had lower education levels, and were more likely to be previous smokers.

Considering the physical activity levels, the median vector magnitude among the sample was 27.19 mg. On average, female (28.61 mg) and younger participants (45–54 yrs., 30.05 mg) showed greater vector magnitude at baseline than the male (27.61 mg) and older counterparts (55-64 yrs., 27.33 mg; 65 yrs. and over, 24.68 mg). The baseline average vector magnitude values were significantly lower among participants who developed AD or VaD (24.25 mg for AD cases; 23.88 mg for VaD cases) compared to non-incident participants (28.18 mg for both non-AD cases and non-VaD cases). In terms of air pollution, individuals diagnosed with either AD or VaD subtypes were exposed to slightly higher levels of exposure to each of the pollutants at baseline compared to non-cases. However, the differences in air pollution exposure levels between case and non-case groups were not statistically significant.

### 3.1 Main effects of physical activity and air pollution

**Figure 2:**
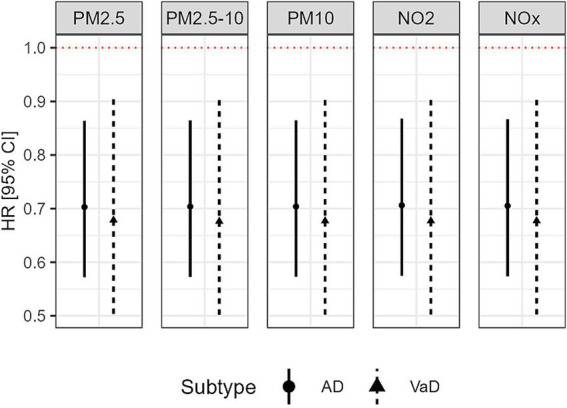
Associations between accelerometer-based physical activity (based on 10.13 mg increment) and incident risks of AD and VaD.

As shown in Table 2, individuals who had one IQR (10.13 mg) higher level of physical activity had a 30% lower risk of being diagnosed with AD (HR = 0.7, 95% CI: 0.57–0.86, *p* < 0.001) and a 32% lower risk of VaD (HR = 0.68, 95% CI: 0.5–0.91, *p* < 0.05) during follow-up, adjusting for covariates. The associations were similar across different models incorporating various pollutants, as shown in Figure 2.

**Table 2:** Main effects of accelerometer-based physical activity and air pollution and risks of incident dementia subtypes.

Variable		AD (*n* = 235)			VaD (*n* = 112)		
		Inciden*t* cases	HR (95% CI)	*p*-value	Incident cases	HR (95% CI)	*p*-value
PM_2.5_	ACC	235	0.7 (0.57–0.86)	<0.001	112	0.68 (0.5–0.91)	<0.05
	Pollution quartile						
	Q1	56	1 [reference]		19	1 [reference]	
	Q2	60	0.99 (0.68–1.44)	0.954	39	1.99 (1.13–3.49)	<0.05
	Q3	54	0.89 (0.6–1.32)	0.564	24	1.27 (0.7–2.33)	0.435
	Q4	65	1.11 (0.74–1.67)	0.621	30	1.53 (0.82–2.86)	0.189
PM_2.5–10_	ACC	235	0.7 (0.57–0.86)	<0.001	112	0.68 (0.5–0.91)	<0.05
	Pollution quartile						
	Q1	58	1 [reference]		19	1 [reference]	
	Q2	53	0.92 (0.62–1.36)	0.682	37	2.13 (1.13–3.99)	<0.05
	Q3	49	0.9 (0.61–1.33)	0.586	26	1.71 (0.89–3.29)	0.113
	Q4	75	1.27 (0.88–1.83)	0.201	30	1.7 (0.85–3.4)	0.14
PM_10_	ACC	235	0.7 (0.57–0.86)	<0.001	235	0.68 (0.5–0.91)	<0.05
	Pollution quartile						
	Q1	62	1 [reference]		18	1 [reference]	
	Q2	58	0.92 (0.64–1.33)	0.671	27	1.51 (0.83–2.76)	0.182
	Q3	44	0.77 (0.51–1.16)	0.212	38	1.94 (1.06–3.55)	<0.05
	Q4	71	1.16 (0.8–1.68)	0.444	29	1.68 (0.9–3.14)	0.106
NO_2_	ACC	235	0.71 (0.57–0.87)	0.001	112	0.68 (0.5–0.91)	<0.05
	Pollution quartile						
	Q1	48	1 [reference]		23	1 [reference]	
	Q2	71	1.46 (1–2.12)	<0.05	45	1.92 (1.16–3.19)	<0.05
	Q3	57	1.25 (0.84–1.84)	0.267	22	0.94 (0.52–1.7)	0.841
	Q4	59	1.35 (0.88–2.06)	0.168	22	0.95 (0.49–1.81)	0.866
NO_x_	ACC	235	0.71 (0.57–0.87)	0.001	112	0.68 (0.5–0.91)	<0.05
	Pollution quartile						
	Q1	57	1 [reference]		25	1 [reference]	
	Q2	66	1.22 (0.85–1.75)	0.293	32	1.28 (0.75–2.17)	0.367
	Q3	52	1.01 (0.69–1.49)	0.948	27	1.14 (0.66–1.98)	0.639
	Q4	60	1.16 (0.78–1.74)	0.464	28	1.21 (0.68–2.18)	0.52

ACC, accelerometer-based total volume of physical activity in mg; AD, Alzheimer’s disease; CI, confidence interval; HR, hazard ratio; NO_2_, nitrogen dioxide; NO_x_, nitrogen oxides; PM, particulate matter; VaD, Vascular Dementia. Cox proportional hazards models were adjusted for age at the initial assessment, sex, race, education, smoking status, BMI, family history of dementias, history of stroke, high blood pressure, and area Townsend deprivation index. Models were estimated for different pollutants separately. HRs for ACC are associated with one IQR increase in ACC values (10.13 mg). The numbers of AD and VaD cases across pollution quartiles differ slightly from those in Table 1, as these results are based on imputation datasets with pollution values imputed.

**Figure 3:**
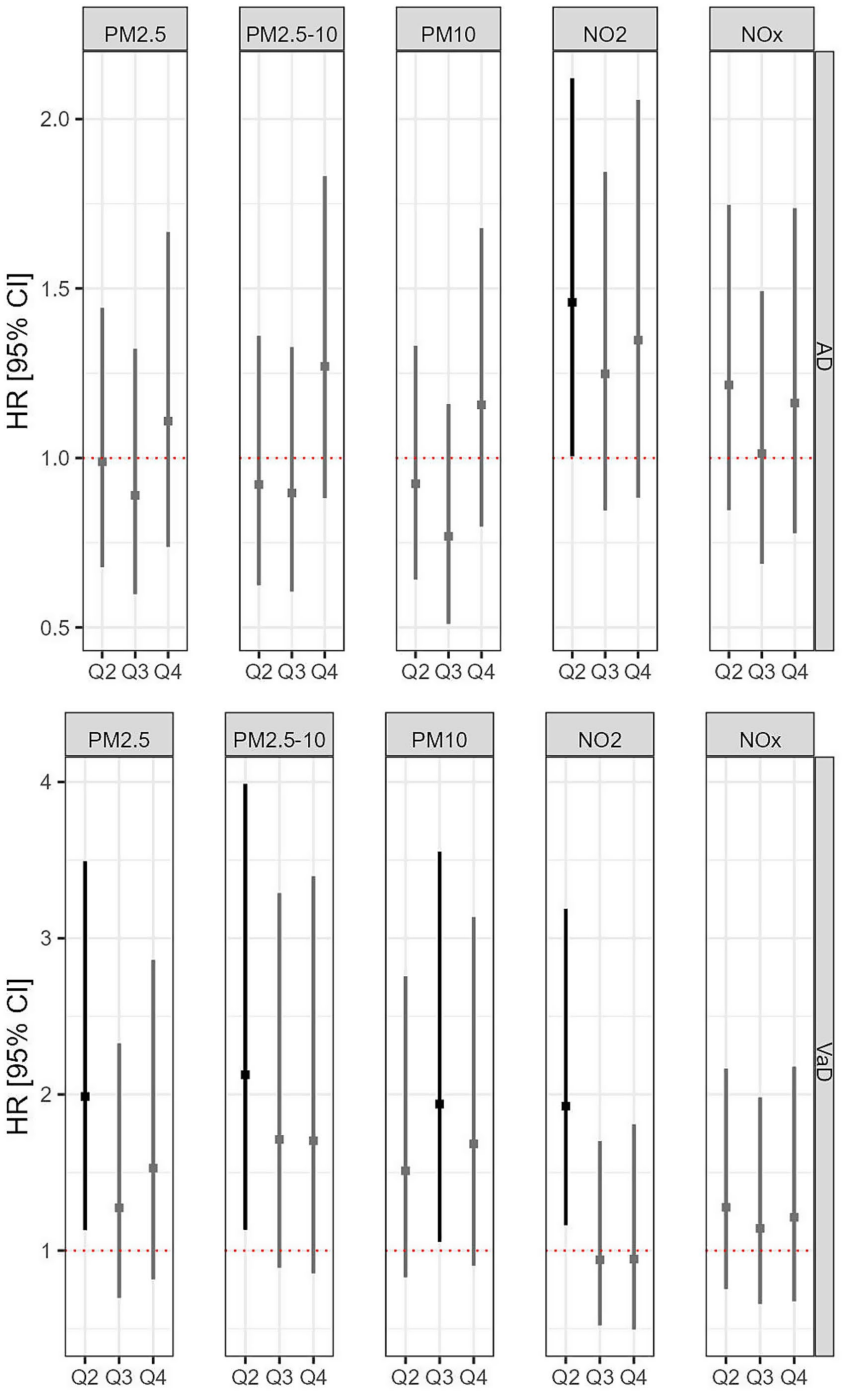
Associations between air pollution and incident risks of AD and VaD.

The associations between air pollution and AD risk showed heterogeneity across pollution types. Compared to those with the lowest NO_2_ exposure (Q1), individuals with higher NO_2_ exposure (Q2) had 46% higher hazard of developing AD (HR = 1.46, 95% CI: 1–2.12, p < 0.05). However, participants who had the greatest exposures (Q3 and Q4) did not show a significantly higher risk of developing AD (Q3 vs. Q1, HR = 1.25, 95% CI: 0.84–1.84, *p* = 0.267; Q4 vs. Q1, HR = 1.35, 95% CI: 0.88–2.06, *p* = 0.168) than those in Q1. Additionally, exposures to PM_2.5_, PM_2.5–10_, PM_10_, and NO_x_ were not significantly associated with the risk of AD. For VaD, the results indicated significant associations between higher air pollution exposures and increased risk of VaD for some groups. Specifically, the HRs were 1.99 (95% CI, 1.13–3.49; *p* < 0.05) for PM_2.5_ (Q2 vs. Q1), 2.13 (95% CI, 1.13–3.99; *p* < 0.05) for PM_2.5–10_ (Q2 vs. Q1), 1.94 (95% CI, 1.06–3.55; *p* < 0.05) for PM_10_ (Q3 vs. Q1), and 1.92 (95% CI, 1.16–3.19; *p* < 0.05) for NO_2_ (Q2 vs. Q1), respectively. NO_x_ exposure did not show a significant association with the risk of VaD, as illustrated in Figure 3. We also estimated fully adjusted single-exposure models (see Supplementary eTable 5). Results were generally consistent with those from models with the main effects of both exposures; however, the associations between PM and VaD appeared stronger in single-exposure models.

### 3.2 Interaction effects between physical activity and air pollution on dementia risk

**Figure 4:**
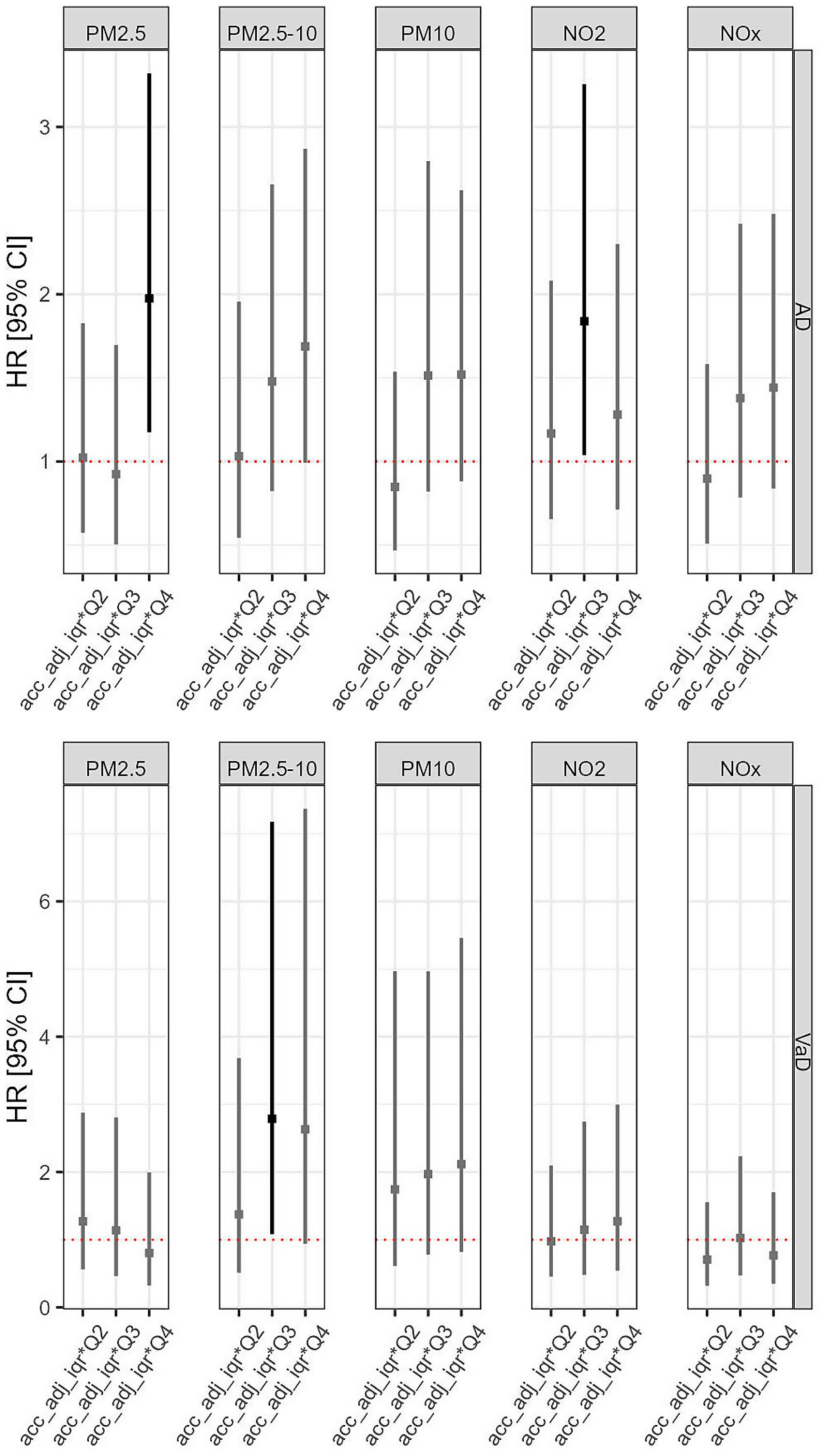
Interactive effects of accelerometer-based physical activity and air pollution on incident risks of AD and VaD.

As shown in Table 3, when including interaction terms between physical activity and all five types of air pollution in the Cox model, the results for most interactions did not reach statistical significance for either AD or VaD. However, the model revealed a significantly higher risk of incident AD associated with the highest level (Q4 vs. Q1) of PM_2.5_ exposure when combined with physical activity (HR = 1.97, 95% CI: 1.17–3.32; *p* < 0.05), as shown in Figure 4. In addition, the interaction between a high level (Q3 vs. Q1) of NO_2_ exposure and physical activity was significantly associated with an increased risk of AD (HR = 1.84, 95% CI: 1.04–3.26; *p* < 0.05). For VaD, a significant interaction effect was found between high PM_2.5–10_ exposure (Q3 vs. Q1) and physical activity, which was associated with an increased risk of VaD (HR = 2.79, 95% CI: 1.08–7.18; *p* < 0.05).

**Table 3:** The interaction effect between accelerometer-based physical activity (ACC, mg) and air pollution and risk of incident dementia.

Pollutant		AD (*n* = 235)		VaD (*n* = 112)	
	Interactions	HR (95% CI)	*p*-value	HR (95% CI)	*p*-value
PM_2.5_					
	ACC *Q1	1 [reference]		1 [reference]	
	ACC *Q2	1.02 (0.57–1.83)	0.937	1.27 (0.56–2.88)	0.566
	ACC *Q3	0.92 (0.5–1.7)	0.8	1.14 (0.46–2.81)	0.777
	ACC *Q4	1.97 (1.17–3.32)	<0.05	0.8 (0.32–2)	0.638
PM_2.5–10_					
	ACC *Q1	1 [reference]		1 [reference]	
	ACC *Q2	1.03 (0.54–1.96)	0.925	1.38 (0.51–3.69)	0.529
	ACC *Q3	1.48 (0.82–2.66)	0.193	2.79 (1.08–7.18)	<0.05
	ACC *Q4	1.69 (0.99–2.87)	0.055	2.63 (0.94–7.37)	0.072
PM_10_					
	ACC *Q1	1 [reference]		1 [reference]	
	ACC *Q2	0.85 (0.47–1.54)	0.588	1.74 (0.61–4.97)	0.303
	ACC *Q3	1.51 (0.82–2.8)	0.188	1.97 (0.78–4.97)	0.155
	ACC *Q4	1.52 (0.88–2.62)	0.135	2.12 (0.82–5.46)	0.125
NO_2_					
	ACC *Q1	1 [reference]		1 [reference]	
	ACC *Q2	1.17 (0.66–2.08)	0.599	0.98 (0.46–2.1)	0.954
	ACC *Q3	1.84 (1.04–3.26)	<0.05	1.15 (0.48–2.75)	0.757
	ACC *Q4	1.28 (0.71–2.3)	0.41	1.27 (0.54–3)	0.58
NO_x_					
	ACC *Q1	1 [reference]		1 [reference]	
	ACC *Q2	0.9 (0.51–1.58)	0.709	0.71 (0.32–1.56)	0.39
	ACC *Q3	1.38 (0.78–2.42)	0.265	1.03 (0.47–2.23)	0.949
	ACC *Q4	1.44 (0.84–2.48)	0.188	0.77 (0.35–1.7)	0.521

ACC, accelerometer based total volume of physical activity in mg; AD, Alzheimer’s disease; BMI, body mass index; Cl, confidence interval; HR, hazard ratio; NO_2_, nitrogen dioxide; NO_x_, nitrogen oxides; PM, particulate matter; SD, standard deviation; VaD, Vascular Dementia. Cox proportional hazards regressions were adjusted for age at the initial assessment, sex, race, education, smoking status, family history of dementias, history of stroke, high blood pressure, Townsend deprivation index, BMI, and main effects of ACC and air pollution.

**Figure 5:**
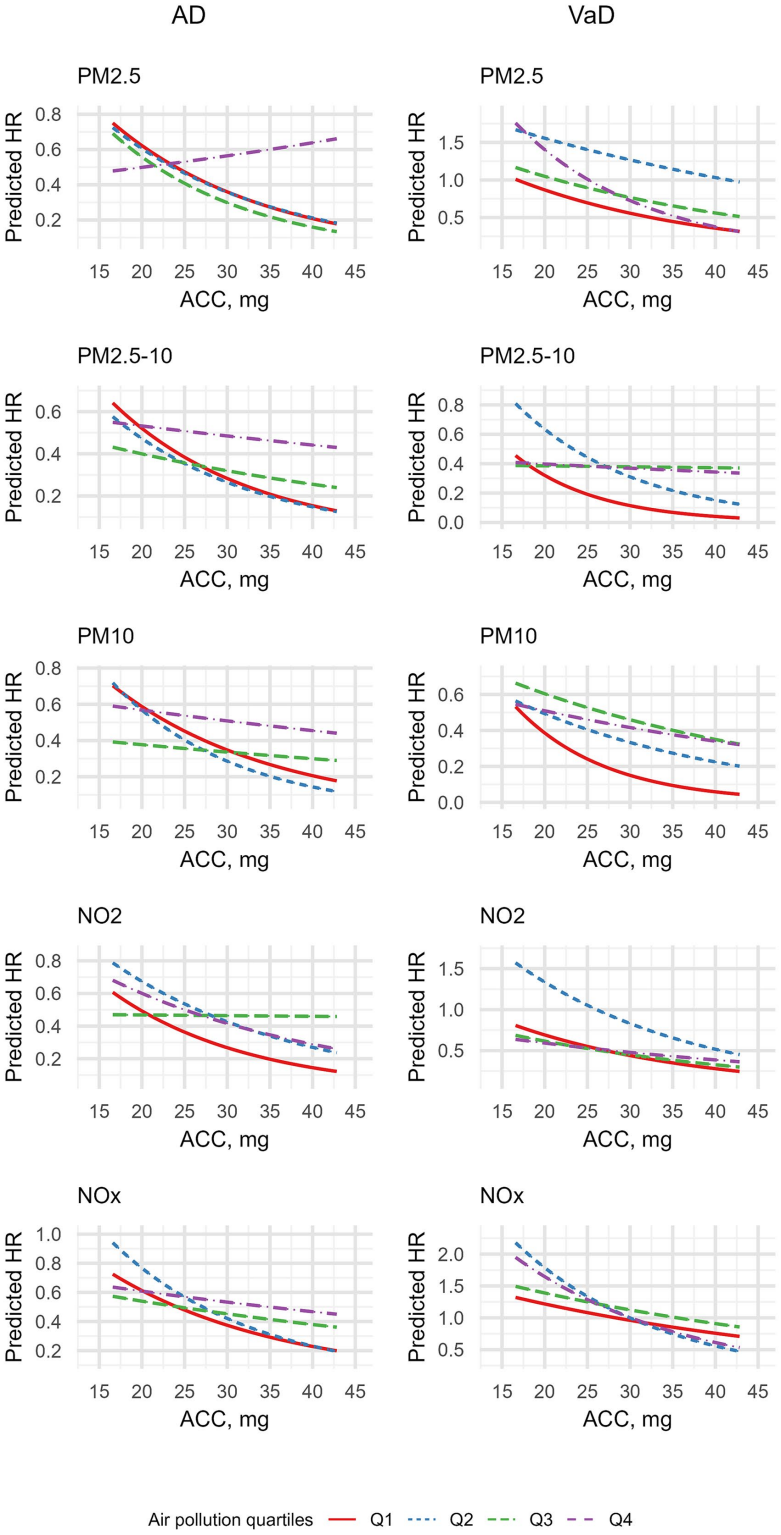
Predicted hazard ratios of incident AD and VaD based on Cox proportional hazards models with interaction terms between physical activity and air pollution and covariates.

We also estimated the predicted HRs at different physical activity levels, stratified by air pollution quartiles, as shown in Figure 5. For most air pollutants, an overall decline in dementia risk was observed with increasing physical activity intensity across all air pollution exposure quartiles for both AD and VaD. However, we found an exception for the highest level (Q4) of PM_2.5_ exposure, where the predicted HR of AD increases along with the increment of physical activity levels.

### 3.3 Sensitivity analysis

We conducted several sensitivity analyses to assess the robustness of results. First, we ran the models using the complete-case dataset, which excluded all missing values of all variables (see Supplementary eFigures 1–3). Second, we excluded dementia cases diagnosed within the first 2 years of the initial assessment to mitigate possible reverse causality (see Supplementary eFigures 4–6). Third, we restricted the analysis to participant’s aged 60 years or older at baseline to explore whether the effects were more pronounced among older individuals (see Supplementary eFigures 7–9).

For physical activity, the prospective effect was consistent across most sensitivity analyses and dementia subtypes, except for participants aged 60 or older, where the effect was not significant in the VaD subtype. Regarding air pollution, the association between NO_2_ exposure and an increased risk of AD was consistent in the analysis of complete cases. Exposures to PM_2.5_, PM_2.5–10_, PM_10_, and NO_x_ remained not significantly associated with the risk of AD across all sensitivity analyses. For VaD, exposure to PM_2.5_, PM_2.5–10_, and PM_10_ was consistently associated with an increased risk, aligning with the main results. However, the association between NO_2_ exposure and VaD risk was significant only in two sensitivity analyses: excluding cases diagnosed within the first 2 years and restricting to participants aged 60 or older. The non-significant association between NO_x_ exposure and VaD risk was consistent across all analyses.

When interaction terms between physical activity and air pollution were included, we observed consistently significant associations in specific scenarios. For AD, excluding cases diagnosed within the first 2 years revealed significant interaction effects between physical activity and PM_2.5_, as well as NO_2_, on increased AD risk. The significant interaction effect between physical activity and NO_2_ was also found in the analysis using participants who were older than 60 years old. No significant interactions were found for PM_2.5–10_, PM_10_, or NO_x_ in relation to AD. For VaD, the interaction between physical activity and PM_2.5–10_ was significantly associated with an increased risk among participants older than 60 years old, while other interactions did not show significant results, consistent with the main findings.

We further estimated the associations between physical activity and dementia risk, stratified by air pollution quartiles, using imputed and complete-case data (see Supplementary eFigures 10, 11). The results generally indicated a stronger protective effect of physical activity against AD and VaD at lower levels of air pollution, though some heterogeneity was observed. Operating both air pollution and physical activity values in quartiles, we found significant interaction effects between exposures to particulate matters and NO_2_ and physical activity on AD risk but not for VaD (see Supplementary eFigure 12).

## 4 Discussion

In this large-scale prospective longitudinal study, we analyzed data from 96,661 participants in the UK Biobank to investigate the association between air pollution, accelerometer-based physical activity, and the risk of dementia subtypes, including AD and VaD. Our findings suggested that a greater physical activity level is protective against both dementia subtypes. These results are consistent with other prospective studies that employ accelerometer-based measures to analyze physical activity and incident dementia risk (17, 52–54). Several mechanisms may explain physical activity’s protective effects against AD and VaD. First, regular physical activity improves cardiovascular and metabolic health by lowering blood pressure and reducing the risk of Type 2 diabetes (55, 56), both of which are established risk factors for dementia (4). Second, physical activity enhances cerebral blood flow, facilitating neurobiological reactions and promoting cerebral angiogenesis (55, 57). This process not only nourishes brain cells and facilitates the clearance of amyloid beta protein, a major pathological feature of AD, but also increases the brain blood supply, potentially reducing the risk of ischemic stroke, which is a primary cause of VaD (55). Third, animal studies have shown that voluntary physical activity increases brain-derived neurotrophic factor and other growth factors, which support brain plasticity and neurogenesis (58). Moreover, it enhances brain resilience to injury and improves cognitive performance in learning and memory (58). Furthermore, studies argued that physical activity played a crucial role in the prevention and management of mental health disorders, particularly depression, which is also a recognized risk factor of dementia (4).

We also found a stronger protective effect of physical activity against incident VaD than AD. This is largely consistent with other epidemiological studies that examine the relationships between physical activity and both AD and VaD. For example, two observational studies based on large prospective cohorts found that physical activity was significantly associated with a reduced risk of VaD but had no effects on AD (9, 59). Animal studies have also shown that physical activity does not appear to protect against AD pathology, such as synaptic proteins PSD-95, synaptophysin, or amyloid- β , in brain issues (59). A few hypotheses have been proposed to explain the potential mechanisms by which physical activity protects against VaD, including increased blood flow to the brain, improved cardiovascular and metabolic health, and enhanced arterial elasticity (55, 60).

Higher exposure to NO_2_ was significantly associated with an increased risk of AD, while higher levels of PM_2.5_, PM_2.5–10_, PM_10_, and NO_2_ were associated with a greater risk of VaD. These associations were observed after adjusting for age, sex, race, education, smoking status, family history of dementia, history of stroke, high blood pressure, Townsend deprivation index, and BMI. Our findings were aligned with an animal study reporting that NO_2_ inhalation accelerated AD-related pathological abnormalities and cognitive dysfunction (61). For VaD, particulate matters may increase the risk of incidence through vascular damage and covert brain infarcts, as previous studies have reported associations between PM_2.5_ exposure and smaller brain volume, as well as between long-term PM_10_ exposure and VaD risk (62, 63).

When incorporating the interaction term between physical activity and air pollution in the model, we found that higher exposure to PM_2.5_ and NO_2_ was significantly associated with an increased risk of incident AD when engaging in physical activity. Additionally, a significant interaction was observed between PM_2.5–10_ exposure and physical activity for VaD, indicating an increased risk of VaD. These findings suggest that the protective effect of physical activity on the risk of dementia subtypes is attenuated by high levels of air pollution exposure, with variations across dementia subtypes and air pollutants. The complexity of this interaction may arise from multiple factors. First, engaging in physical activity increases minute ventilation, potentially leading to greater inhalation and deposition of particulate matter in the respiratory system and bloodstream (64), which may partially explain why high air pollution levels attenuate the protective benefits of physical activity against AD and VaD. Second, individuals may modify their physical activity behaviors in response to high levels of air pollution to minimize exposure. Previous studies suggest that ambient air pollution can negatively impact physical activity levels (65–67). For example, a nationwide study in the US found that higher exposure to PM_2.5_ was significantly associated with lower odds of engaging in physical activity (65). This may partially explain why the significant negative impact of air pollution was observed mostly in Q2 rather than in Q3 or Q4 in the main models. However, this hypothesis cannot be tested using current data. Future studies that leverage longitudinal measures of air pollution and physical activity may elucidate the influence of air pollution on changes in physical activity patterns to better understand their joint effects on dementia risk. Furthermore, the varied size of air pollutant particles and their implications for biological processes underlying dementia subtypes and upstream risk factors, may partially explain the inconsistency in interaction effects on dementia subtypes. Previous studies suggest that fine particulate matter such as PM_2.5_ can deposit deeply into the alveolar region and enter the bloodstream, causing systemic oxidative stress that affects the generation and accumulation of AD-inducing amyloid beta protein (68, 69). In contrast, coarser PM_2.5–10_ is primarily deposited in the upper respiratory tract, where it may exacerbate conditions like asthma, which is significantly associated with the risk of stroke, a major risk factor of VaD (69–71).

In our study, we used accelerometer-based measures of physical activity, which were not subject to self-reporting biases as they rely on participants’ actual physical movements rather than subjective recall. In addition to accelerometers, objective measures of physical activity used in related studies include heart rate monitoring and pedometers (72–74). In contrast, subjective measures, such as self-reported questionnaires, are more susceptible to recall bias (75) and social desirability bias (76, 77), which may lead to over- or under-estimation of actual activity levels. Furthermore, even among studies using subjective physical activity measures, there is considerable variability in effect sizes. In contrast, these objective measures, such as accelerometers, provide real-time and unbiased data, allowing for a more reliable estimation of physical activity levels. In our study, we found that individuals with one IQR (10.13 mg) higher level of physical activity experienced a 30% lower risk of being diagnosed with AD (HR = 0.7, 95% CI: 0.57–0.86, *p* < 0.001) and a 32% lower risk of VaD (HR = 0.68, 95% CI: 0.5–0.91, *p* < 0.05).

There are several limitations in this study. First, air pollution exposure assessments were based on 2010 estimates only, which may not fully capture air pollution exposure over time and its cumulative impact on dementia risk. Second, air pollution exposure was assessed based on residential locations, which may not reflect exposure at other locations, such as work or recreation. However, it has been shown that residential and time-activity integrated air pollution exposures show high concordance in epidemiological studies (78), indicating relatively low bias in air pollution exposure assessment based on residential locations alone. Third, the accelerometer data lack information on whether the activity occurred indoors or outdoors. Future research should capture both spatial and temporal variations in air pollution exposure and differentiate between indoor and outdoor physical activities to better understand their combined effects on dementia risk. Fourth, the numbers of AD and VaD cases are small, therefore, the sample may not be sufficiently powered to detect significant interactive effects. Fifth, due to power limitations, we applied single-pollution models in our analysis. A multi-pollutant approach may more accurately estimate the independent effects of each pollution exposure, as well as their interactions with physical activity, on dementia risk. Sixth, we adjusted for a range of sociodemographic, behavioral, and environmental confounders in our regression models. However, missing confounder bias (e.g., hearing loss, green space) may still be present. Seventh, the UK Biobank study participants are not population-representative and are selected toward healthier individuals (79, 80). There is further evidence that participants in the accelerometer study were more likely to be female, White British, and socioeconomically advantaged compared to the overall biobank sample (81). Given these potential selection biases, the results in our study may not be generalizable to the general population. And lastly, a validation study of algorithmically defined dementia outcomes in the UK Biobank showed that the positive predictive values (PPV) for AD and VaD were 71.4 and 43.8%, respectively (82). The relatively low PPV for VaD may have contributed to the inconsistency in our findings, as many VaD cases may not have been correctly identified through data linkage.

## 5 Conclusion

Based on accelerometer-derived measures, higher levels of physical activity are associated with a lower risk of both incident AD and VaD, and the effect is stronger for VaD. Significant moderating effects of air pollution on the associations between physical activity and incident risk of dementia subtypes are observed for PM_2.5_ and NO_2_ for AD and PM_2.5–10_ for VaD. Public health initiatives may consider the synergistic effects of physical activity and air quality to reduce dementia risk at the population scale.

## Data Availability

The data analyzed in this study is subject to the following licenses/restrictions: The data that support the findings of this study are available from the UK Biobank. Data are available from the UK Biobank after application approval. Requests to access these datasets should be directed to https://www.ukbiobank.ac.uk.

## Footnotes

1
http://www.escapeproject.eu/

2
http://www.sahsu.org/

3
http://www.bioshare.eu/
