# Supplementary material for: Accelerometer-based physical activity, air pollution and risk of dementia subtypes: a prospective study using UK biobank
Source: Front Public Health. 2026 Jan 13;13:1695462. doi: 10.3389/fpubh.2025.1695462 (PMC12835275; doi:10.3389/fpubh.2025.1695462)
Supplement: Supplementary file 1 [file Table_1.DOCX]

Appendix

# Content

| Page |  |
| --- | --- |
| 2 | eTable 1. Codes used in the UK Biobank study to identify dementia in Alzheimer’s disease cases. |
| 2 | eTable 2. Codes used in the UK Biobank study to identify vascular dementia cases. |
| 3 | eTable 3. Definitions of variables in the imputation models. |
| 4 | eTable 4. Variables entered in the imputation models. |
| 5 | eTable 5. Associations between air pollution and accelerometer-based physical activity and incident risk of dementia subtypes from single exposure models. |
| 6 | eFigure 1. Main effects of physical activity based on complete cases analysis. |
| 7 | eFigure 2. Main effects of air pollution based on complete cases analysis. |
| 8 | eFigure 3. Interactive effects of air pollution and physical activity based on complete cases analysis. |
| 9 | eFigure 4. Main effects of physical activity based on the subsample after excluding dementia cases within the first two years of follow-up. |
| 10 | eFigure 5. Main effects of air pollution based on the subsample after excluding dementia cases within the first two years of follow-up. |
| 11 | eFigure 6. Interactive effects of air pollution and physical activity based on the subsample after excluding dementia cases within the first two years of follow-up. |
| 12 | eFigure 7. Main effects of physical activity based on the subsample of participants aged 60 years and over at baseline. |
| 13 | eFigure 8. Main effects of air pollution based on the subsample of participants aged 60 years and over at baseline. |
| 14 | eFigure 9. Interactive effects of air pollution and physical activity based on the subsample of participants aged 60 years and over at baseline. |
| 15 | eFigure 10. Associations between physical activity and dementia subtype risks stratified by air pollution level, based on imputation data. |
| 16 | eFigure 11. Associations between physical activity and dementia subtype risks stratified by air pollution level, based on complete cases analysis. |
| 17 | eFigure 12. Interactive effects of air pollution and physical activity (both quartiles) on dementia subtype risks. |

**eTable 1. Codes used in the UK Biobank study to identify dementia in Alzheimer’s disease cases**

| Code Type | Code | Code Text |
| --- | --- | --- |
| ICD 9 Code | 331.0 | Alzheimer’s disease |
| ICD 10 Code | F00 | Dementia in Alzheimer’s disease |
| ICD 10 Code | F00.0 | Dementia in Alzheimer’s disease with early onset |
| ICD 10 Code | F00.1 | Dementia in Alzheimer’s disease with late onset |
| ICD 10 Code | F00.2 | Dementia in Alzheimer’s disease, atypical or mixed type |
| ICD 10 Code | F00.9 | Dementia in Alzheimer’s disease, unspecified |
| ICD 10 Code | G30 | Alzheimer’s disease |
| ICD 10 Code | G30.0 | Alzheimer’s disease with early onset |
| ICD 10 Code | G30.1 | Alzheimer’s disease with late onset |
| ICD 10 Code | G30.8 | Other Alzheimer’s disease |
| ICD 10 Code | G30.9 | Alzheimer’s disease unspecified |

**eTable 2. Codes used in the UK Biobank study to identify vascular dementia cases.**

| Code Type | Code | Code Text |
| --- | --- | --- |
| ICD 9 Code | 290.4 | Arteriosclerotic dementia |
| ICD 10 Code | F01 | Vascular dementia |
| ICD 10 Code | F01.0 | Vascular dementia of acute onset |
| ICD 10 Code | F01.1 | Multi-infarct dementia |
| ICD 10 Code | F01.2 | Subcortical vascular dementia |
| ICD 10 Code | F01.3 | Mixed cortical and sub-cortical vascular dementia |
| ICD 10 Code | F01.8 | Other vascular dementia |
| ICD 10 Code | F01.9 | Vascular dementia, unspecified |
| ICD 10 Code | I67.3 | Binswanger’s disease |

**Multiple imputation**

Under the multiple imputation with chained equations algorithm for multiple imputation, missing values of each partially observed variable is imputed in turn based on observed data and imputed values of other variables in the imputation model. As researchers have noted, for analyses containing interactions, passive imputation (or ‘‘impute then transform’’) for interaction terms can create bias (specifically, the effect estimates of the effects of each of the variables in the interaction will bias away from the null and the estimate for the interaction term will bias towards the null) and under-coverage of confidence intervals. This is because imputation fails to take into account the fact that the effect of one independent variable in the interaction on the dependent variable may be different depending on the values of the other independent variable in the interaction.(1) To reduce biases in estimates of effects and confidence intervals, we included air pollution, physical activity, as well as their interaction term in the imputation models. Multiple imputation was carried out for each pollutant (PM_2·5_, PM_2·5-10_, PM_10_, NO_2_ and NO_x_) separately.

**eTable 3. Definitions of variables in the imputation models.**

| Variable | Data field | Definition | Values | Type |
| --- | --- | --- | --- | --- |
| Age | 21022 | Age at initial assessment |  | Continuous |
| Sex | 31 | Sex | Male, Female | Binary |
| Race/ethnicity | 21000 | Race/ethnicity | Recoded to be White, Black, Asian, Mixed or other | Categorical |
| Education | 6138 | Qualification | Recoded to be College or higher, Less than college | Binary |
| Smoking status | 20116 | Smoking status of the participant | Current, Previous, Never | Categorical |
| Family history of dementia |  | Parent or sibling ever had dementia diagnosis | Yes, No | Binary |
| Stroke history | 6150 | Ever told by a doctor that they have had a stroke | Yes, No | Binary |
| High blood pressure (HBP) | 6150 | Ever told by a doctor that they have had high blood pressure | Yes, No | Binary |
| Adjusted average  acceleration | 90087 | No-wear time bias adjusted average acceleration |  | Continuous |
| Townsend deprivation index | 22189 | Index for area level socioeconomic deprivation. |  | Continuous |
| PM_2·5_ | 24006 | PM_2·5_ in 2010 | Divided into quartiles (Q1-Q4) | Categorical |
| PM_2·5-10_ | 24008 | PM_2·5-10_ in 2010 | Divided into quartiles (Q1-Q4) | Categorical |
| PM_10_ | 24005 | PM_10_ in 2010 | Divided into quartiles (Q1-Q4) | Categorical |
| NO_2_ | 24003 | NO_2_ in 2010 | Divided into quartiles (Q1-Q4) | Categorical |
| NO_x_ | 24004 | NO_x_ in 2010 | Divided into quartiles (Q1-Q4) | Categorical |
| Days to first occurrence of AD or VaD | 53,  42020,  42022 | Days between date of initial assessment (field 53), date of first occurrence of AD (field 42020), and date of first occurrence of VaD (field 42022) |  | Continuous |

**eTable 4. Variables entered in the imputation models.**

| Age (continuous) | Sex (binary) | Race/ethnicity (categorical) |
| --- | --- | --- |
| Education (binary) | Smoking status (categorical) | Family history of dementia (binary) |
| Stroke history (binary) | High blood pressure (binary) | Townsend deprivation index (continuous) |
| Adjusted average  acceleration (continuous) | Air pollution (PM, NO_2_ or NO_x_) | Adjusted average acceleration*pollutant |
| Days to first occurrence of AD or VaD (continuous, in days) |  |  |

**eTable 5.** Associations between air pollution and accelerometer-based physical activity (ACC) and incident risk of dementia subtypes from single exposure models.

|  | | **AD** | **VaD** |
| --- | --- | --- | --- |
|  |  | HR (95% CI) | HR (95% CI) |
| ACC, mg | | 0.63 (0.49-0.82)** | 0.41 (0.27-0.64)*** |
| PM_2.5_ | Q1 (ref) | 1 | 1 |
|  | Q2 | 1.25 (0.78-2.02) | 2.94 (1.3-6.64)* |
|  | Q3 | 1.32 (0.81-2.13) | 2.44 (1.04-5.74)* |
|  | Q4 | 1.6 (0.98-2.62) | 2.52 (1.01-6.26)* |
| PM_2.5-10_ | Q1 (ref) | 1 | 1 |
|  | Q2 | 0.89 (0.55-1.43) | 2.65 (1.17-6.03)* |
|  | Q3 | 0.92 (0.57-1.48) | 2.52 (1.08-5.87)* |
|  | Q4 | 1.41 (0.91-2.18) | 2.03 (0.85-4.87) |
| PM_10_ | Q1 (ref) | 1 | 1 |
|  | Q2 | 1.2 (0.76-1.88) | 2.84 (1.26-6.43)* |
|  | Q3 | 0.85 (0.51-1.39) | 2.31 (0.97-5.49) |
|  | Q4 | 1.34 (0.85-2.11) | 2.33 (0.98-5.57) |
| NO_2_ | Q1 (ref) | 1 | 1 |
|  | Q2 | 1.47 (0.9-2.39) | 1.87 (0.96-3.62) |
|  | Q3 | 1.62 (1-2.62) | 1.02 (0.47-2.22) |
|  | Q4 | 1.74 (1.02-2.94)* | 0.92 (0.37-2.29) |
| NO_x_ | Q1 (ref) | 1 | 1 |
|  | Q2 | 1.3 (0.82-2.05) | 1.27 (0.64-2.49) |
|  | Q3 | 1.03 (0.63-1.69) | 0.95 (0.44-2.02) |
|  | Q4 | 1.48 (0.9-2.42) | 1.22 (0.55-2.7) |

Abbreviations: ACC: accelerometer-based physical activity; AD: Alzheimer’s disease; HR, hazard ratio; NO_2_: Nitrogen dioxide; NO_x_, Nitrogen oxide; PM: particulate matter; VaD: Vascular dementia.

Note: Coefficient estimates are generated from single-exposure models, indicating the associations between ACC or air pollution and incident risk of AD or VaD. All models are adjusted for age, sex, race/ethnicity, education, smoking status, family history of dementia, stroke history, high blood pressure, and area Townsend Deprivation Index. Coefficients for ACC are hazard ratios for one interquartile range increase in ACC (10.13 mg).

* P<0.05; ** P<0.01; *** P<0.001


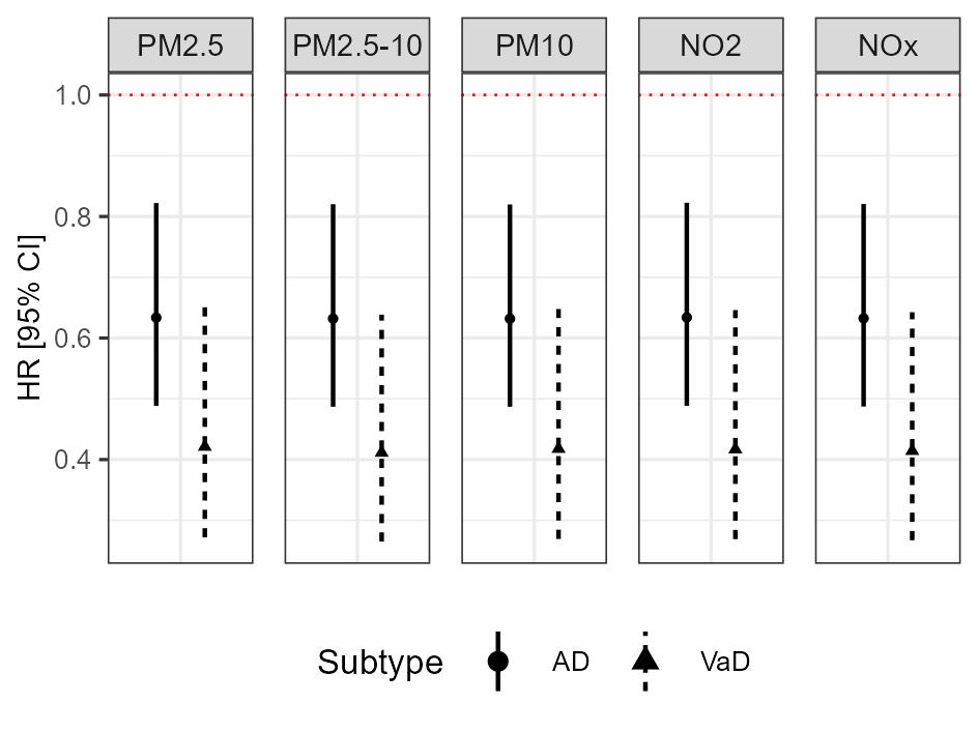


**eFigure 1.** Main effects of physical activity based on complete cases analysis. HRs are associated with one interquartile range increase in accelerometer-derived physical activity measure. All models are adjusted for age, sex, race/ethnicity, education, smoking status, family history of dementia, stroke history, high blood pressure, area Townsend Deprivation Index, and air pollution (quartiles).


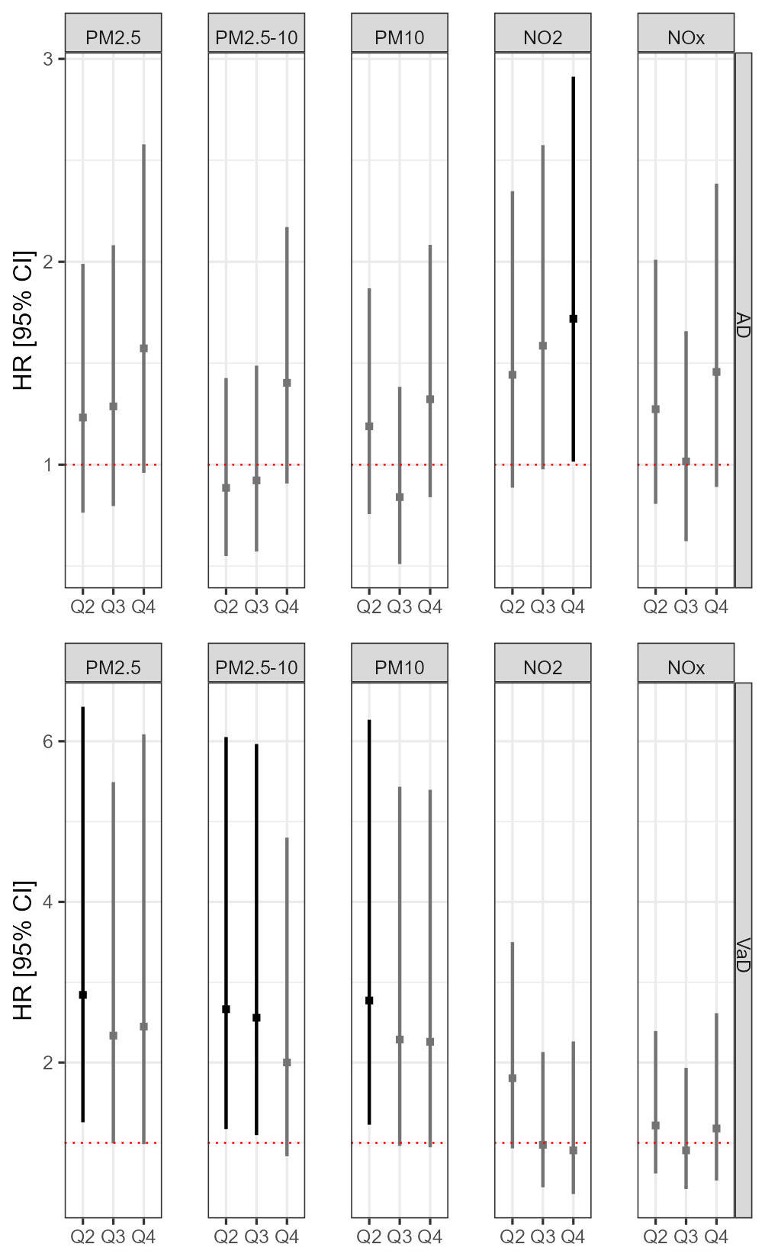


**eFigure 2.** Main effects of air pollution based on complete cases analysis. HRs are associated with different quartiles of air pollution with the first quartile as the reference group. All models are adjusted for age, sex, race/ethnicity, education, smoking status, family history of dementia, stroke history, high blood pressure, area Townsend Deprivation Index, and physical activity.

**
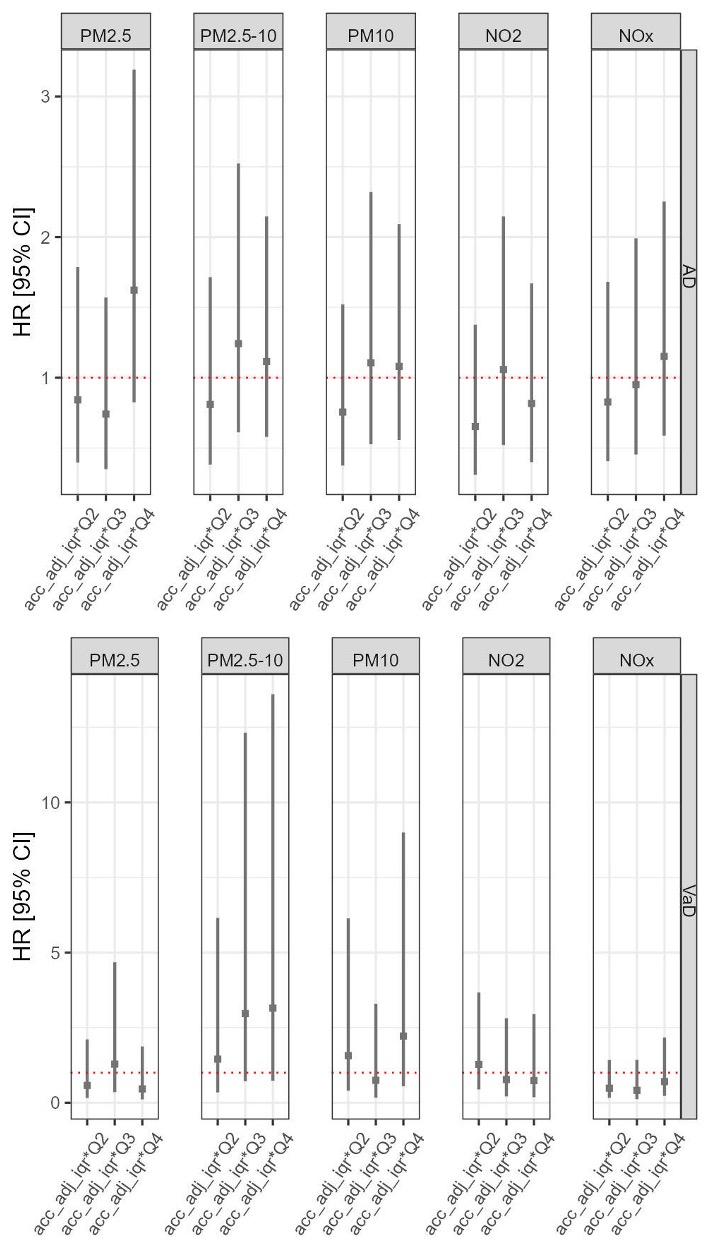
**

**eFigure 3.** Interactive effects of air pollution and physical activity based on complete cases analysis. HRs are presented for interaction terms with the first quartile of air pollution*physical activity (IQR) as the reference group. All models are adjusted for age, sex, race/ethnicity, education, smoking status, family history of dementia, stroke history, high blood pressure, and area Townsend Deprivation Index.

**
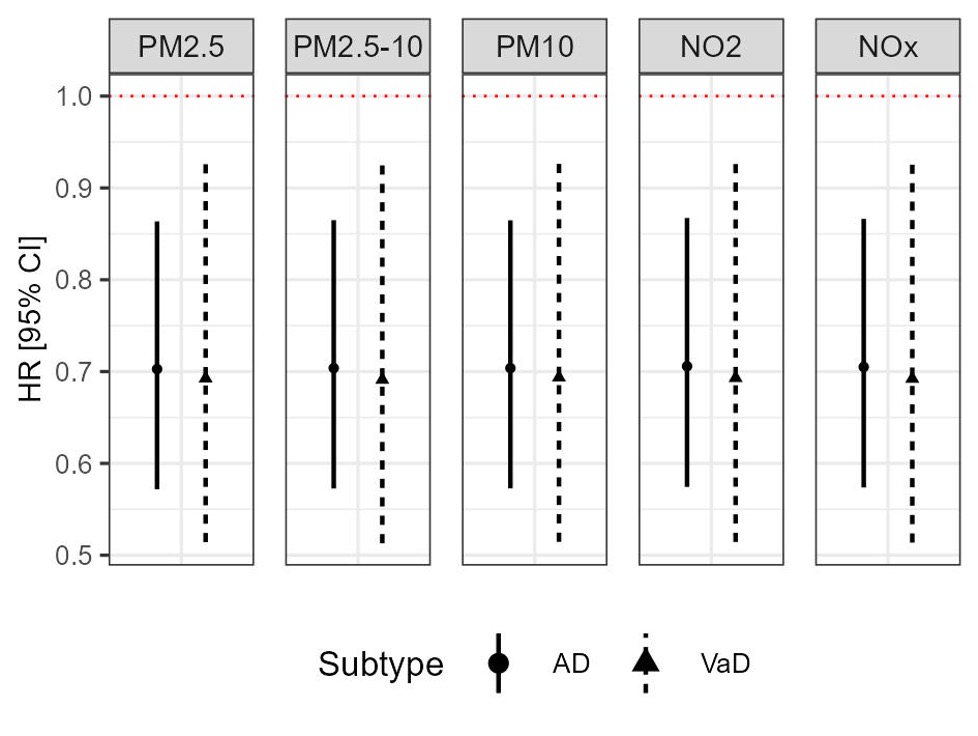
**

**eFigure 4.** Main effects of physical activity based on the subsample after excluding dementia cases within the first two years of follow-up. HRs are associated with one interquartile range increase in accelerometer-derived physical activity measure. All models are adjusted for age, sex, race/ethnicity, education, smoking status, family history of dementia, stroke history, high blood pressure, area Townsend Deprivation Index, and air pollution (quartiles).


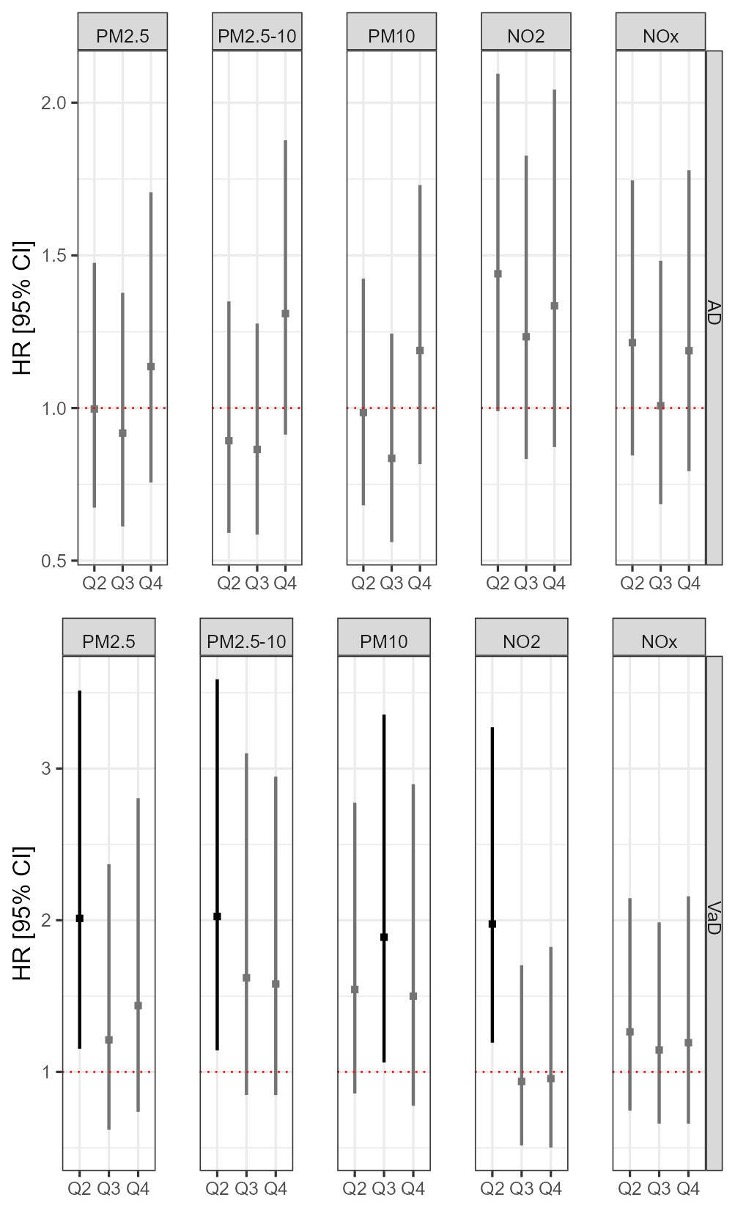


**eFigure 5**. Main effects of air pollution based on the subsample after excluding dementia cases within the first two years of follow-up. HRs are associated with different air pollution quartiles, with the first quartile as the reference group. All models are adjusted for age, sex, race/ethnicity, education, smoking status, family history of dementia, stroke history, high blood pressure, area Townsend Deprivation Index, and physical activity.


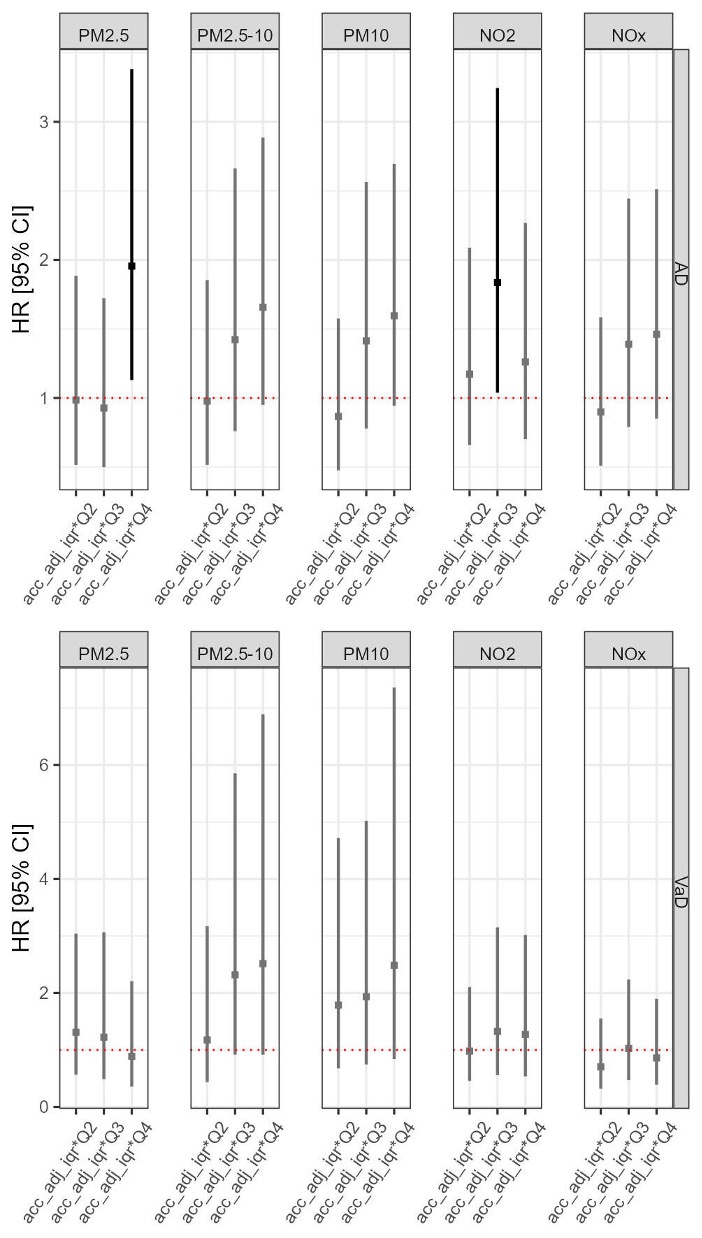


**eFigure 6.** Interactive effects of air pollution and physical activity based on the subsample after excluding dementia cases within the first two years of follow-up. HRs are presented for interaction terms with the first quartile of air pollution*physical activity (IQR) as the reference group. All models are adjusted for age, sex, race/ethnicity, education, smoking status, family history of dementia, stroke history, high blood pressure, and area Townsend Deprivation Index.

**
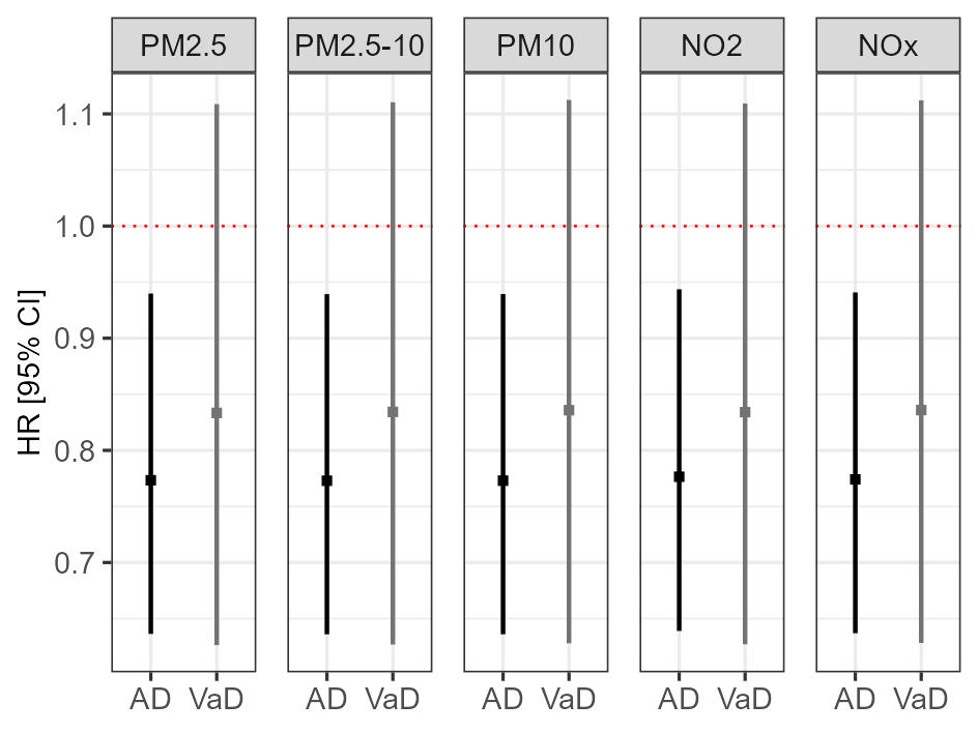
**

**eFigure 7.** Main effects of physical activity based on the subsample of participants aged 60 years and over at baseline. HRs are associated with a one interquartile range increase in accelerometer-derived physical activity measure. All models are adjusted for age, sex, race/ethnicity, education, smoking status, family history of dementia, stroke history, high blood pressure, area Townsend Deprivation Index, and air pollution (quartiles).


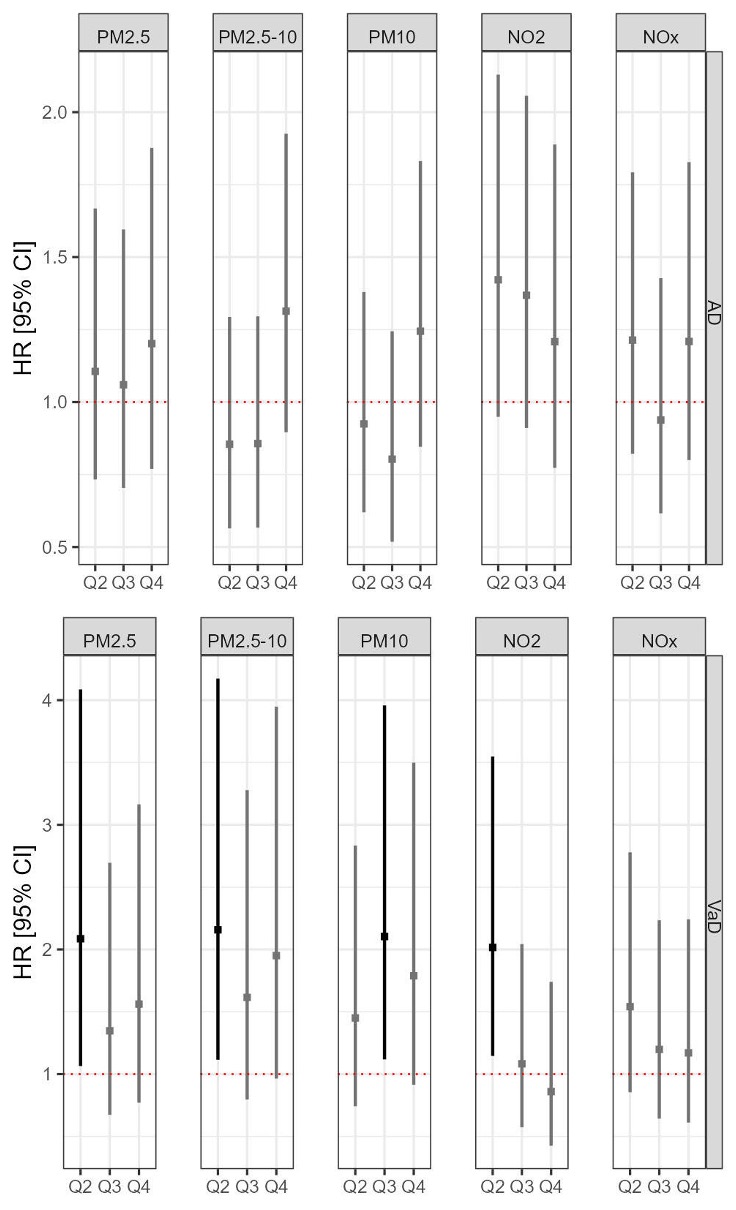


**eFigure 8.** Main effects of air pollution based on the subsample of participants aged 60 years and over at baseline. HRs are associated with different air pollution quartiles, with the first quartile as the reference group. All models are adjusted for age, sex, race/ethnicity, education, smoking status, family history of dementia, stroke history, high blood pressure, area Townsend Deprivation Index, and physical activity.

**
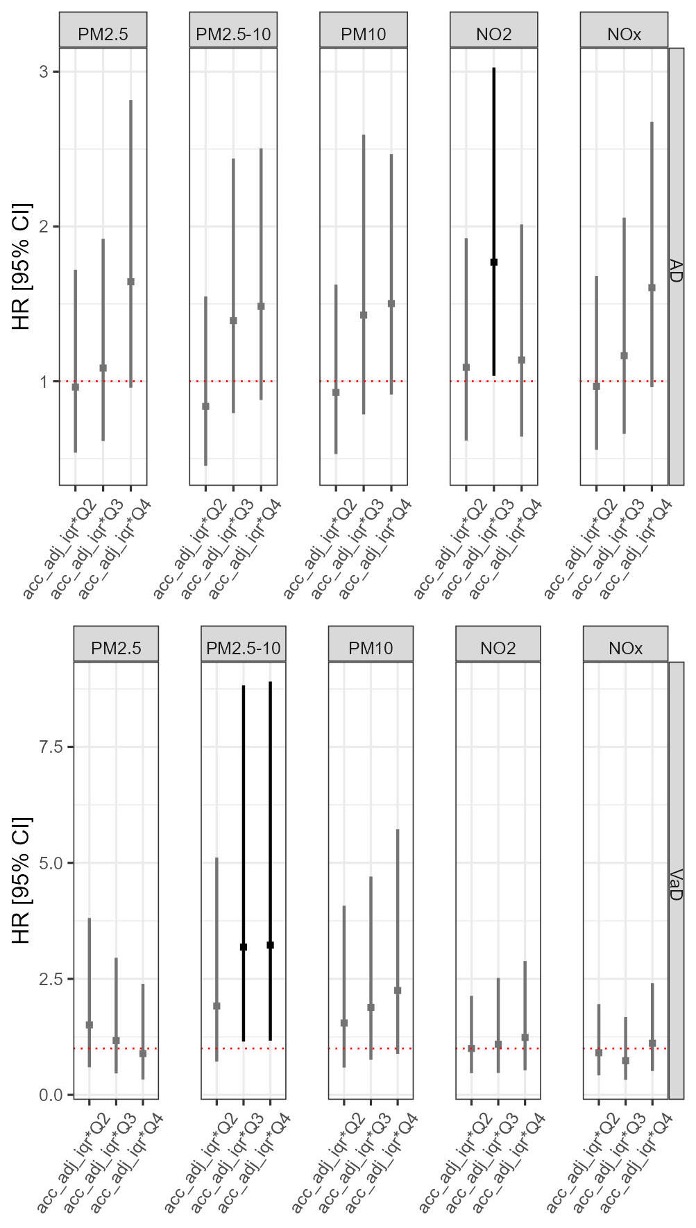
**

**eFigure 9.** Interactive effects of air pollution and physical activity based on the subsample of participants aged 60 years and over at baseline. HRs are presented for interaction terms with the first quartile of air pollution*physical activity (IQR) as the reference group. All models are adjusted for age, sex, race/ethnicity, education, smoking status, family history of dementia, stroke history, high blood pressure, and area Townsend Deprivation Index.

**
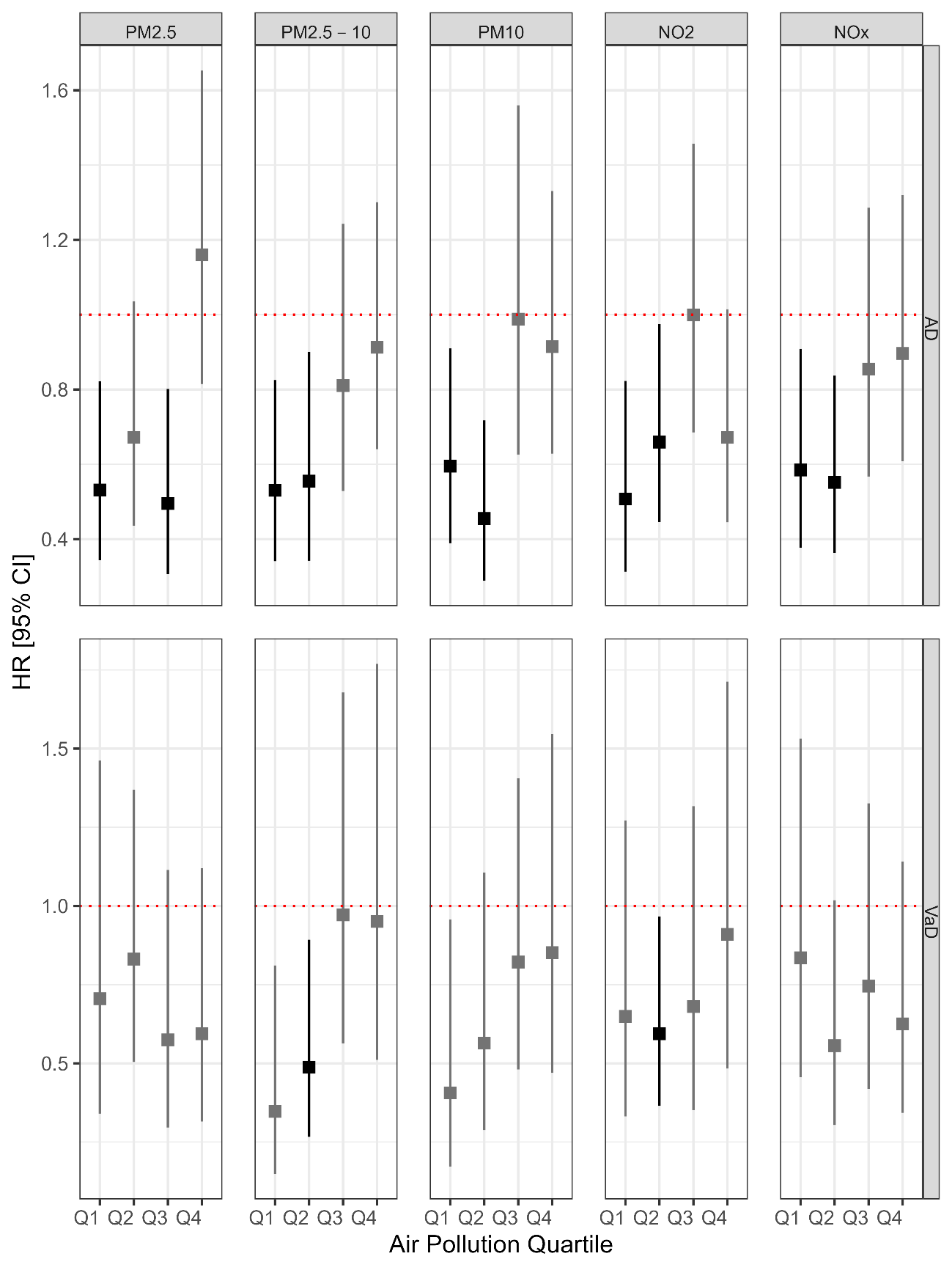
**

**eFigure 10.** Associations between physical activity and dementia subtype risks stratified by air pollution level, adjusted for covariates, based on imputation data. The hazard ratios (HRs) are associated with a one interquartile range increase in accelerometer-derived physical activity measure.


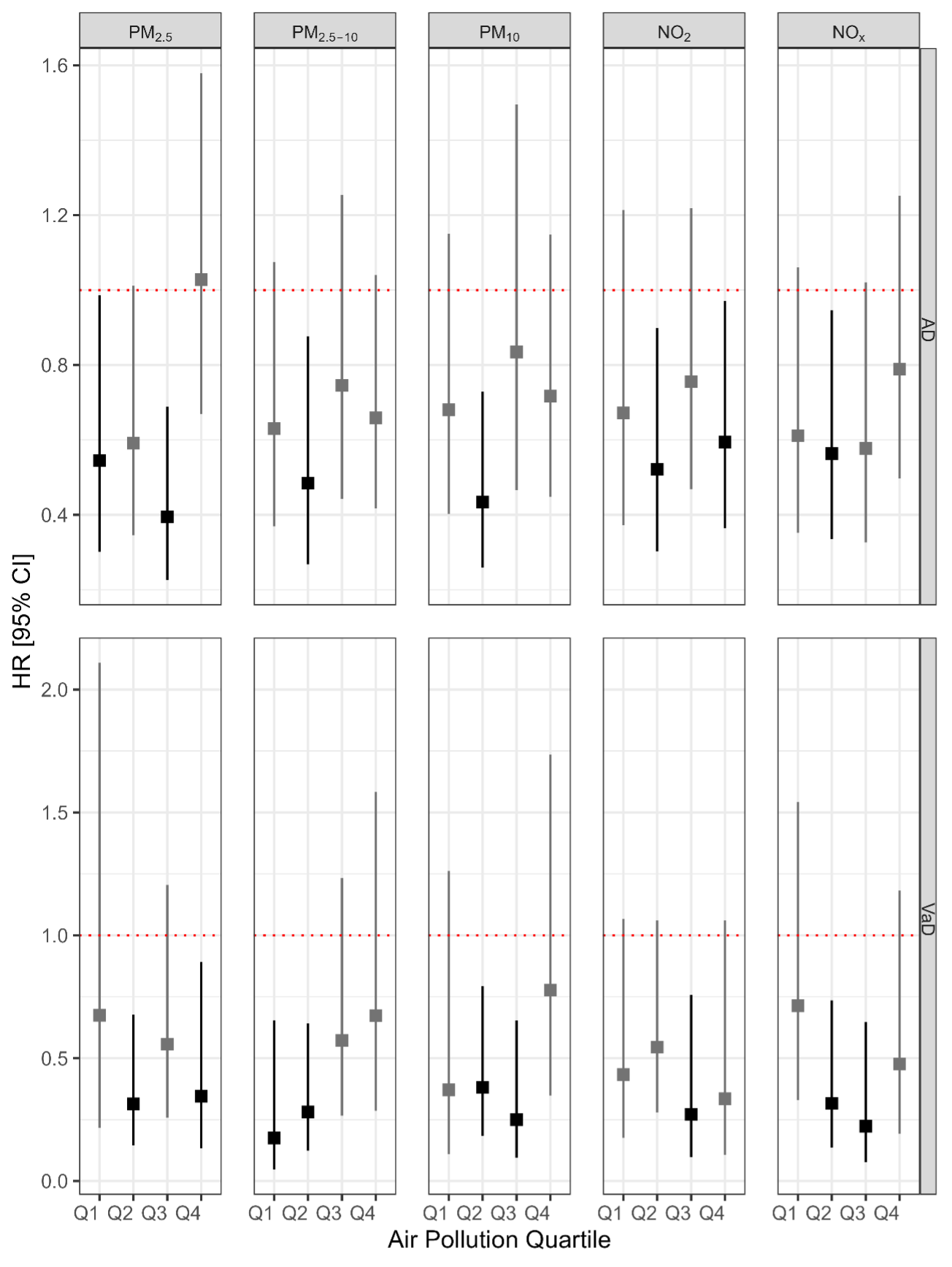


**eFigure 11.** Associations between physical activity and dementia subtype risks stratified by air pollution level, adjusted for covariates, based on complete cases analysis. The hazard ratios (HRs) are associated with a one interquartile range increase in accelerometer-derived physical activity measure.


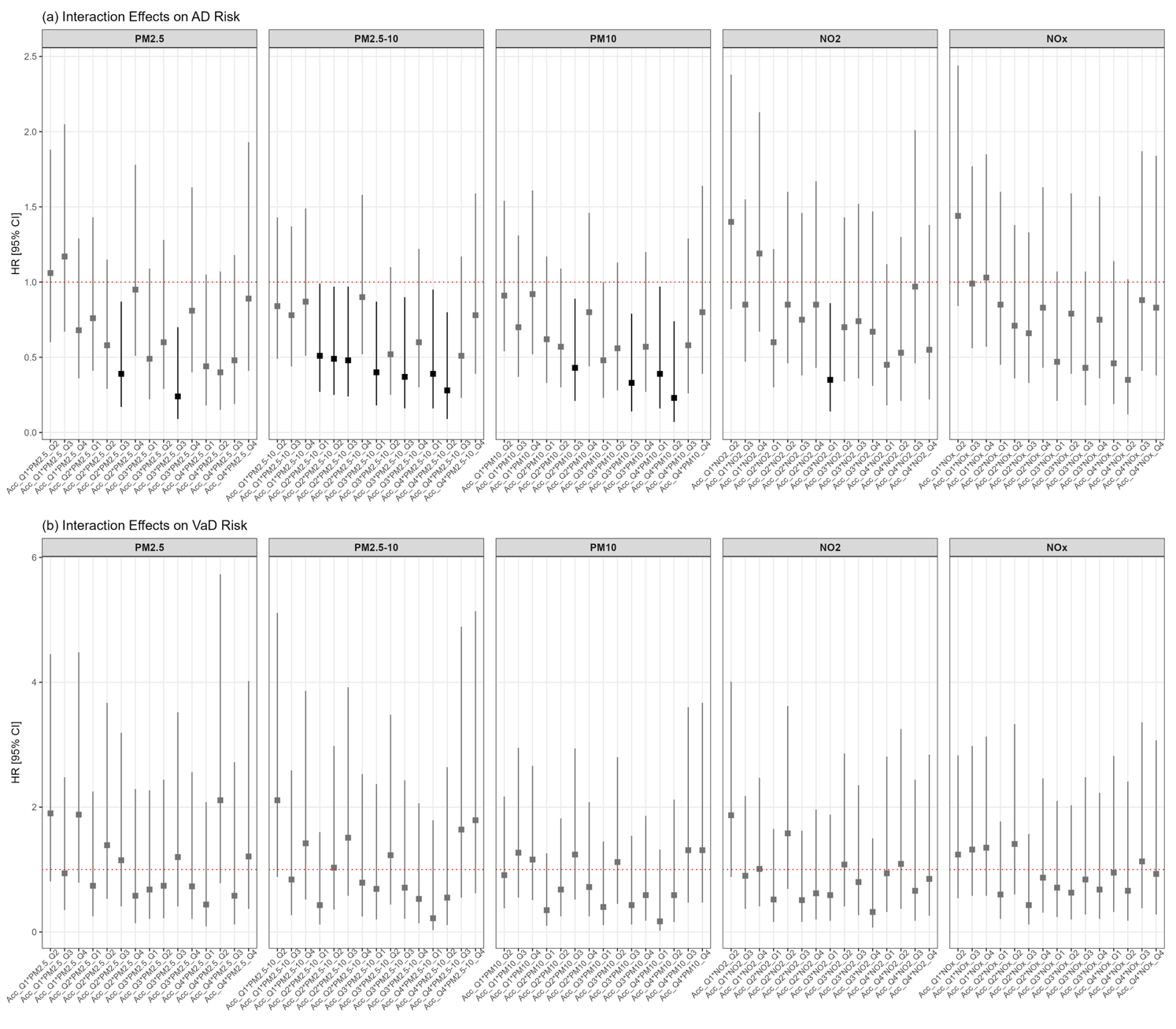


**eFigure 12.** Interactive effects of air pollution and physical activity (both operated as quartiles) on dementia subtype risks. Coefficient estimates of the interaction terms are presented. All models are adjusted for age, sex, race/ethnicity, education, smoking status, family history of dementia, stroke history, high blood pressure, and area Townsend Deprivation Index, and the main effects of air pollution and physical activity.
